# Data on the soil and vegetation properties at the small gully catchment area: Steppe region of Kalmykia Republic (South Russia)

**DOI:** 10.1016/j.dib.2021.107746

**Published:** 2021-12-24

**Authors:** Ivan N. Semenkov, Maria V. Konyushkova, Galya V. Klink, Victoria V. Krupskaya, Polina R. Enchilik, Nina M. Novikova

**Affiliations:** a Lomonosov Moscow State University, Leninskie gory st. 1, Moscow 119991, Russia; b Institute for Information Transmission Problems Kharkevich Institute of the Russian Academy of Sciences, Bolshoy Karetny per. 19-1, Moscow 127051, Russia; c Institute of Geology of Ore Deposits, Petrography, Mineralogy and Geochemistry of the Russian Academy of Sciences, Staromonetny per. 35, Moscow 119017, Russia; d Water Problems Institute of the Russian Academy of Sciences, Gubkina st. 3, Moscow 119333, Russia

**Keywords:** Heavy metals, Semi-arid landscapes, Salt-effected soils, Partitioning, Fractionation, Black soils, Plant communities, Above ground phytomass

## Abstract

In rural areas, research on the environment in native (untaught) soils is important to understand the rate of pedogenesis and to prevent the problems associated with hidden huger. In this article, original data on vegetation, chemical properties and elemental and mineralogical composition of Kastanozems (Protosalic, Siltic) and Hypersalic Solonetz (Siltic) of the small gully catchment (2 ha in total) located at the NE Ergeni Upland (Western Kalmykia, Russia) were presented. Vegetation was described and cut off (to characterize an aboveground biomass) at 13 key plots of 1 × 1 m. The list of species of the small gully catchment area amounts to 23 species (predominantly, perennial herbs) belonging to 13 families and 11 orders. The main dominants *are Artemisia lerchiana, A. austriaca, Festuca valesiaca* and *Poa bulbosa*. Soils were described and sampled in 11 cross-sections and two key plots (0 – 10 cm topsoil sampling). In soil water extracts (79 samples in total), electrical conductivity (EC) and pH were measured. In soil samples, particle size distribution, soil organic carbon and CaCO_3_ contents, total concentration of all the macro elements, some trace (Cl, Nb, Rb, Th, Y, Zr) and potentially toxic elements (As, Co, Cr, Cu, Ni, Pb, Sr, V, and Zn) were described. Moreover, the concentration of three mobile fractions of elements (Li, Be, B, Na, Mg, Al, Si, P, S, K, Ca, Ti, V, Cr, Mn, Fe, Co, Ni, Cu, Zn, Sr, Ba, Cd, Pb) measured using Inductively Coupled Plasma Atomic Emission Spectrometry (AES-ICP) was presented. Geochemical indexes of weathering (R – Silica/Alumina, CIW – Chemical Index of Weathering, CIA - Chemical Index of Alteration, WIP – Weathering Index of Parker, PWI –Product of Weathering Index, Vogt Ratio, PIA – Plagioclase Index of Alteration, STI – Silica-Titanium Index, B/A – Bases/Alumina, B/R – Bases/R_2_O_3_, Si/R - Silica/R_2_O_3_, Weathering indexes WI-1 and WI-2, Si/Ses – Silica/Sesquioxides, Si/Fe – Silica/Iron, a – Potassium/Sodium, ba-1 – (Potassium-Sodium)/Alumina, ba-2 – (Calcium-Magnesium)/Alumina, Ba – (Potassium-Sodium-Calcium)/Alumina) were calculated. In 12 bulk soil samples from Kastanozems and Solonetz, mineralogy (X-Ray diffractometry, the Rietveld full-pattern fitting method for quantitative analysis) was described. Data obtained can be used for more confident identification of pollution sources and pollutants’ migration routes, as well as for more effective land-use management, calculating the required doses of nutrients and for adaptation of land use.

## Specifications Table

**Table:** 

Subject	Environmental science (General)
Specific subject area	Environmental Chemistry, Earth Sciences, Biology, Soil Science, Botany, Mineralogy
Type of data	Table Image Chart Graph Figure
How data were acquired	Particle-size distribution was measured using an ‘Analysette 22 Nano Tech’ equipment (Germany). Data on pH-value of a 1:2.5 water extract was acquired using ‘Expert-001’ (Russia). Data on electrical conductivity of a 1:5 water extract was acquired using ‘Expert-002’ (Russia). Soil organic carbon content was measured by a dichromate method [1]. Total content of chemical elements was measured via an Axios X-Ray fluorescence spectrometer made by PANalytical (Netherlands). Data on elemental composition of acetate buffer and 1M nitric acid extracts was acquired using an atomic emission mass-spectrometer ‘iCAP-6500’ by Thermo Scientific (USA). Data on mineralogy was acquired using an ULTIMA-IV X-Ray diffractometer made by Rigaku (Japan) with Cu radiation and a DTex/Ultra semiconductor detector. Phylogenetic tree of species found at the key site was built on a basis of a phylogenetic tree using scripts and instructions from Qian and Jin (2016).
Data format	Raw Analyzed
Parameters for data collection	Data were collected during field and laboratory works. At 13 key plots of 1 × 1 m, vegetation was described. A total of 79 soil samples were collected from the 11 cross-sections and 2 key plots (Fig. 1) at the NE Ergeni Upland (Western Kalmykia, Russia). The Latin names of plant species were given according to (Cherepanov, 1995).
Description of data collection	An aboveground biomass was collected from 11 key plots of 1 × 1 m. Soil samples of 500 – 700 g of a dry weight were collected from a depth 0 – 130 cm (A, B and C soil horizons of Kastanozems and Solonetz).
Data source location	Institution: Lomonosov Moscow State UniversityRegion: Kalmykia RepublicCountry: RussiaThe sampling area is located at the NE Ergeni Upland [5], near the Arshan-Zel'men experimental station [6], [7]. GPS coordinates of the key plots (Fig. 1): 1. N 47.56730° E 44.2979° 2. N 47.56728° E 44.29778° 3. N 47.56735° E 44.29754° 4. N 47.56737° E 44.29751° 5. N 47.56765° E 44.29760° 6. N 47.56774° E 44.29765° 7. N 47.56779° E 44.29767° 8. N 47.56787° E 44.29770° 9. N 47.56793° E 44.29772° 10. N 47.56808° E 44.29779° 11. N 47.56833° E 44.29734° 12. N 47.56769° E 44.29743° 13. NN 47.56769° E 44.29753° Primary data sources for the construction of the phylogenetic tree of species found at the key site were taken from Qian and Jin (2016).
Data accessibility	Repository name: Sadovoe Direct URL to data: https://data.mendeley.com/datasets/t6ky82f87h/2
Related research article	I. Semenkov, M. Konyushkova, Geochemical partition of chemical elements in Kastanozems and Solonetz at the Northeast Ergeni Upland, Russia, Catena. (2022) 105869. 10.1016/j.catena.2021.105869

## Value of the Data

• Data could be used for the assessment of floristic richness variation in semi-arid ecosystems effected by climate change, as well as for mitigation of negative consequences resulted from soil salinity effects.
• Data may be useful for i. farmers and practitioners to adapt and mitigate negative effects formed in semi-arid lands due to salinity and ground water level changes, as well as climate change, ii. for soil scientists to evaluate migration and transformation of substances (including a mineral soil matrix) in neutral and alkaline conditions and iii. for botanist to characterize the flora in steppe regions.
• Data will be important for further estimation of co-evolution of soils and plants in semi-arid regions effected by climate change, human impact and fires.

## 1 Data Description

**Fig. 1:**
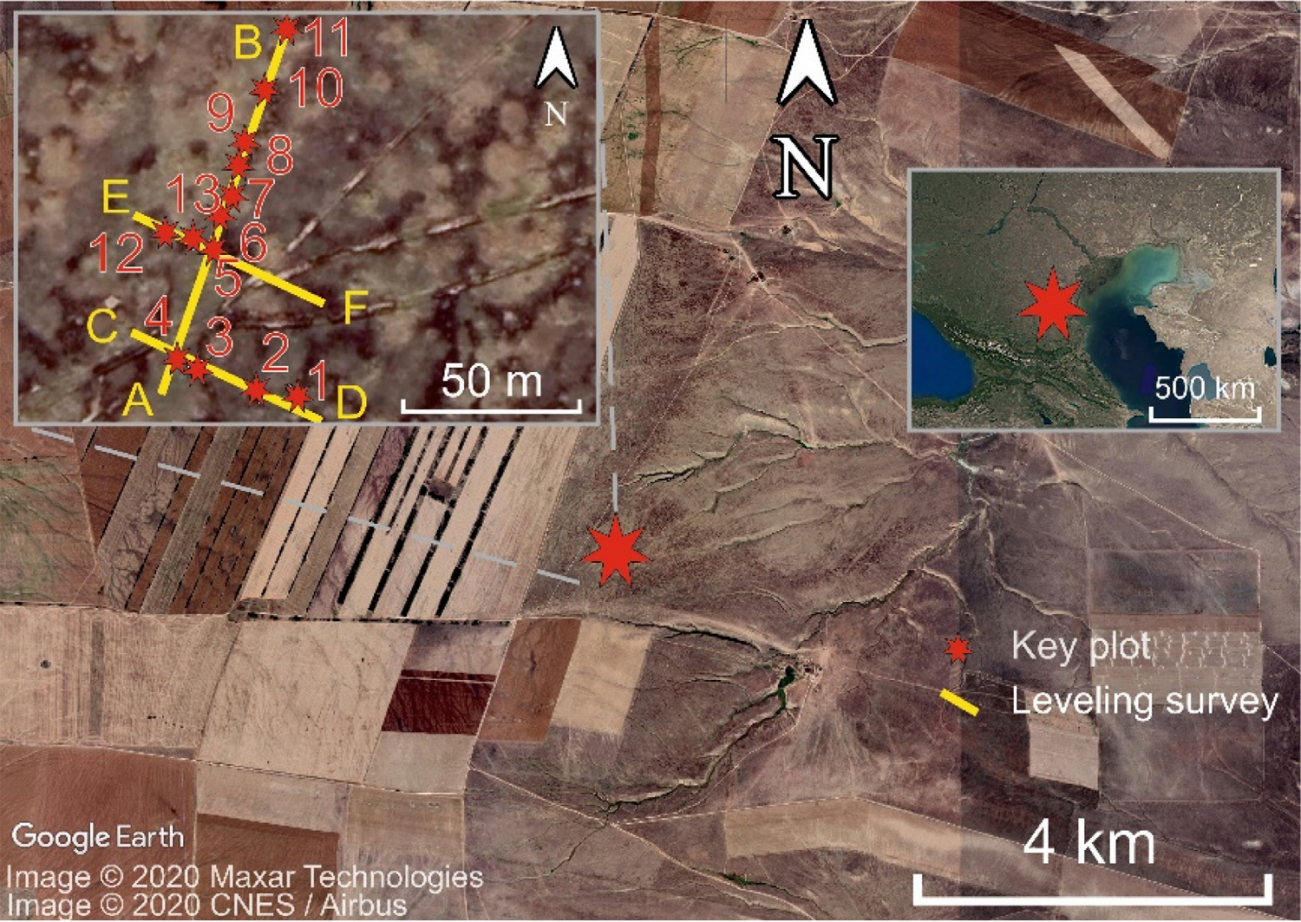
Location of the key plots in the study area situated at the steppe region of Kalmykia Republic (South Russia).

Data were collected at the small gully catchment (2 ha in total) located at the NE Ergeni Upland (Western Kalmykia, Russia) (Fig. 1).

**Fig. 2:**
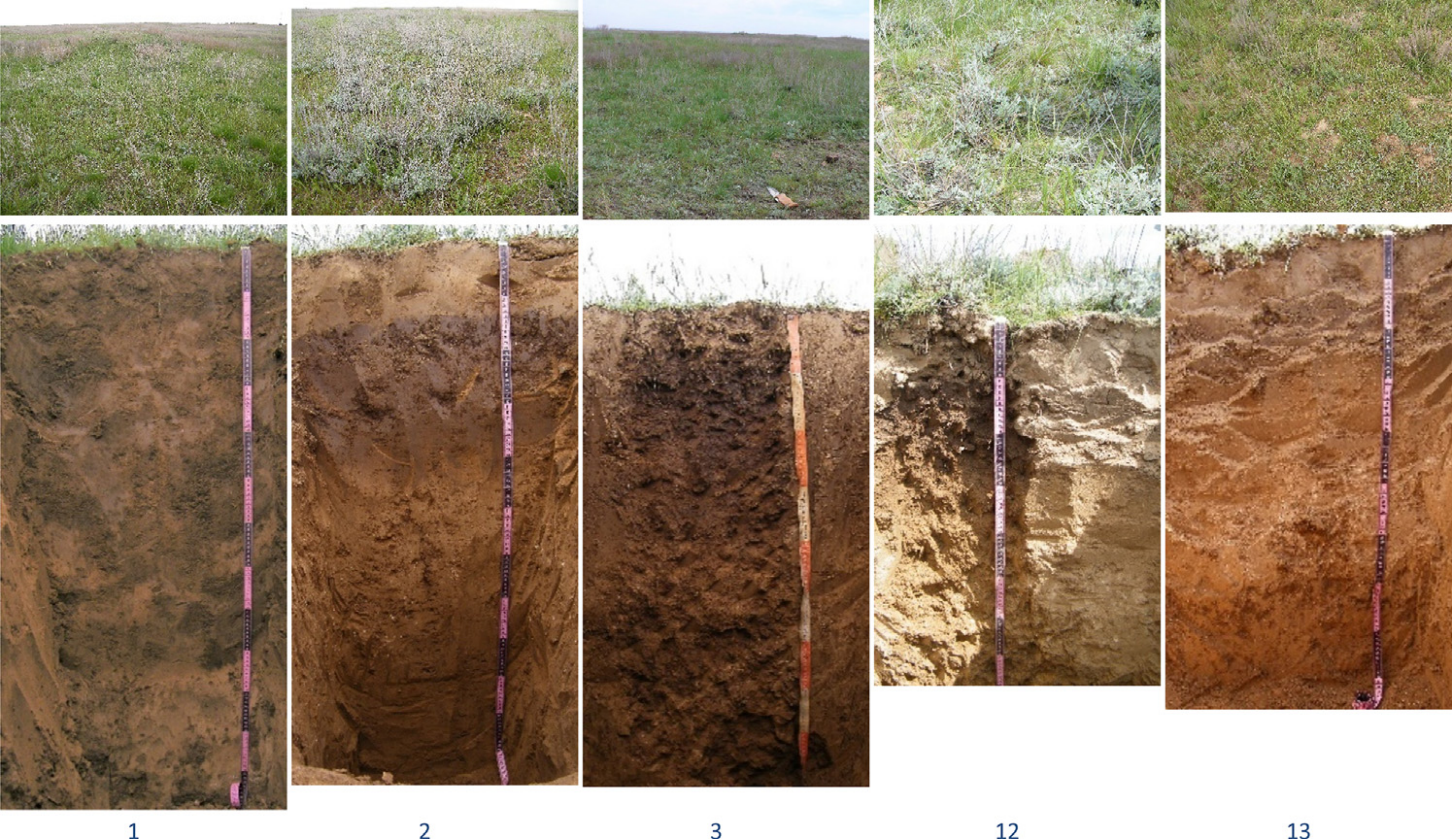
Plants (upper photos) and soils (lower photos) of key plots (1, 3 and 13 – Kastanozems, 2 and 12 – Solonetz) located at the gully interfluve (see Fig. 1).

**Fig. 3:**
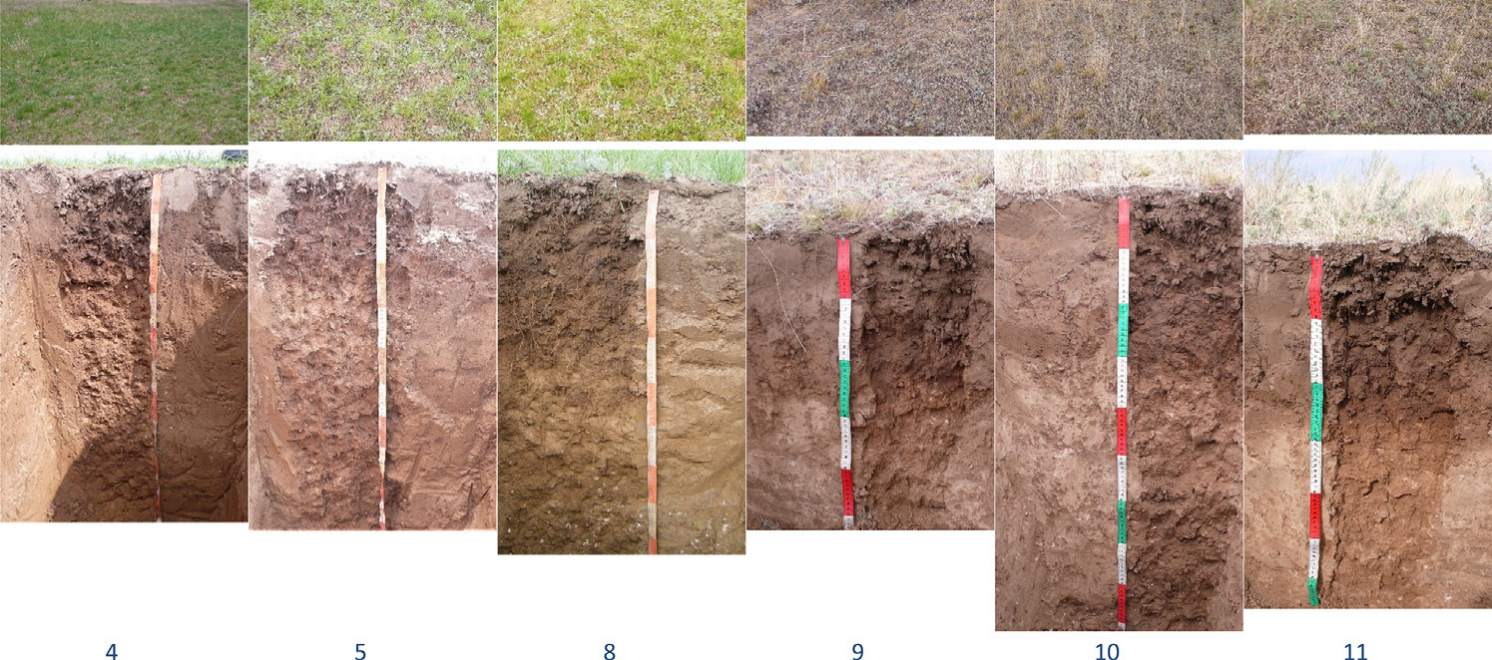
Plants (upper photos) and soils (lower photos) of key plots (4, 5, 8, 10 and 11 – Kastanozems and 9 – Solonetz) located at the gully bottom (see Fig. 1).

**Fig. 4:**
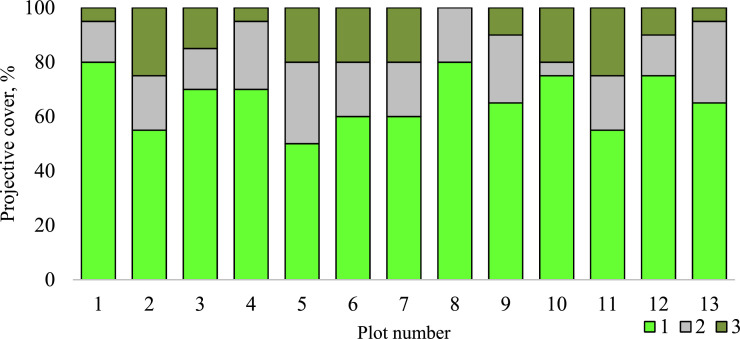
Projective cover of 1 – vascular plants, 2 – bare ground and 3 – mosses and lichens at the plots described at the small gully catchment area.

In the small gully catchment, key plots were accomodated at the interfluve position (Fig. 2) and at the gully bottom (Fig. 3) to characterize plants grown on Kastanozems and Solonetz and (physico-)chemical and mineralogical composition of soils. In this paper, data on the geobotanical descriptions at the 13 key plots of 1 × 1 m (1 m^2^; Table 1, Fig. 4) and data on 79 soil samples collected from 11 cross-sections and 2 key plots where topsoil was sampled were presented.

**Table 1:** List of vascular plant species that are involved in the community occurred in the gully catchment area located at the NE Ergeni Upland.

No	Family and species
	**Family Poaceae Barnhart.**
1	*Agropyron desertorum* (Fisch. ex Link) Schult*.*
2	*Festuca valesiaca* Gaudin
3	*Poa bulbosa* L.
4	*Stipa capillata* L.
5	*Stipa lessingiana* Trin. & Rupr.
6	*Stipa sareptana* A. Beck
	**Family Asteraceae Dumort.**
7	*Artemisia lerchiana* Web*.*
8	*Artemisia pauciflora* Web*.*
9	*Artemisia santonica* L.
10	*Artemisia austriaca* Jacq*.*
11	*Tanacetum achilleifolium* Bieb. Sch.Bip.
	**Family Cyperaceae Juss.**
12	*Carex stenophylla* Wahlenb*.*
	**Family Amaryllidaceae J.St.-Hil.**
13	*Gagea bulbifera* (Pall.) Salisb.
14	*Allium sp.*
	**Family Polygonaceae Juss.**
15	*Polygonum patulum* Bieb*.*
	**Family Amaranthaceae Juss.**
16	*Petrosimonia triandra* (Pall.) Simonk.
	**Family Caryophyllaceae Juss.**
17	*Cerastium sp.*
	**Family Ranunculaceae Juss.**
18	*Ceratocephala testiculata* (Crantz) Bess.
	**Family Brassicaceae Burnett.**
19	*Erophila verna* (L.) Bess.
	**Family Geraniaceae L.**
20	*Geranium sp.*
	**Family Fabaceae Lindhl.**
21	*Astragalus sp.*
	**Family Boraginaceae Juss.**
22	*Lappula squarrosa* (Retz.) Dumort
23	*Myosotis sp.*

**Fig. 5:**
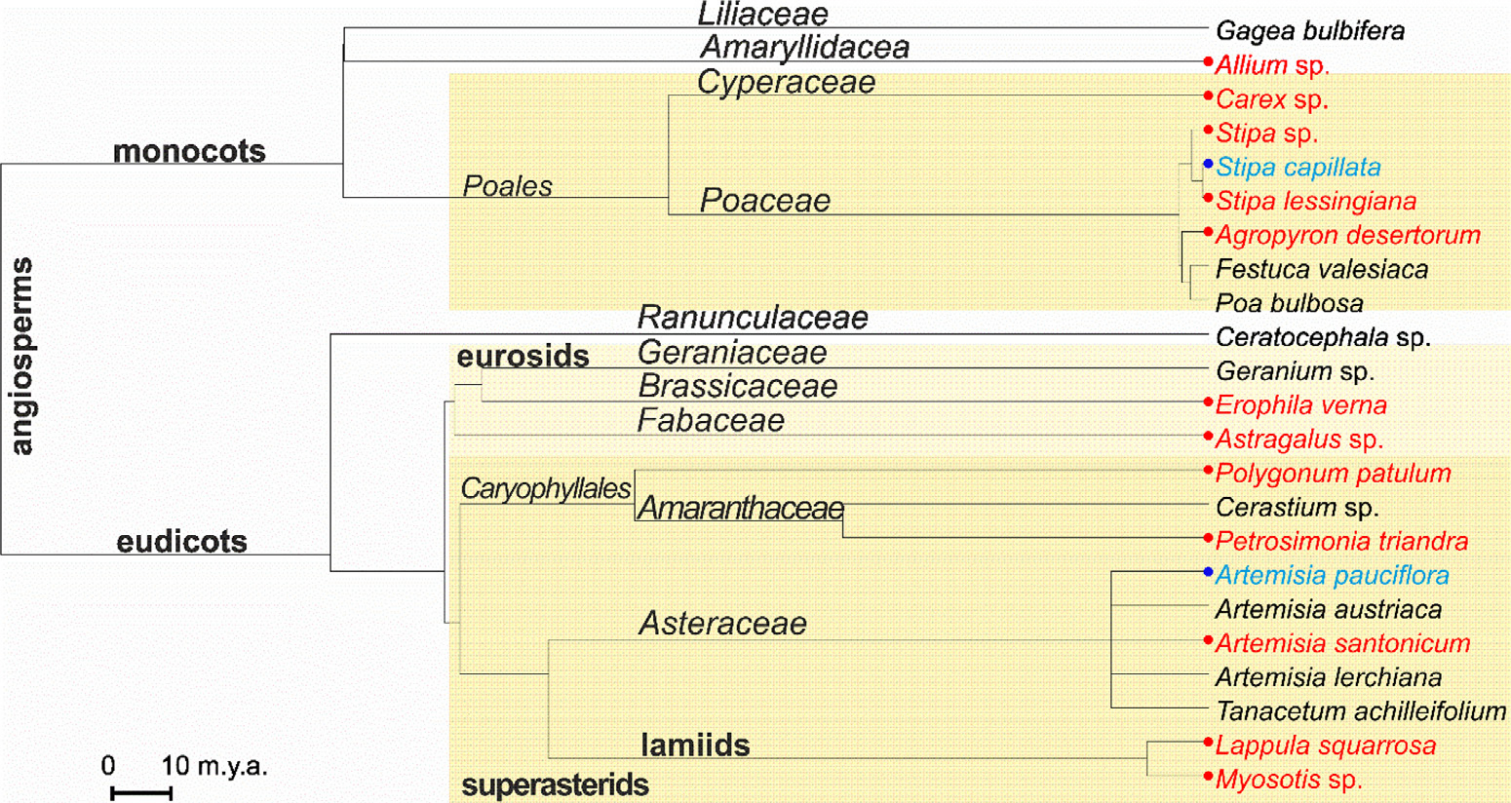
Evolutionary tree of vascular plant species from the small gully catchment area occurred at the NE Ergeni Upland. Species occurred only on Kastanozems are marked in red. Species found only on Solonetz are marked blue; m.y.a. – million years ago.

23 species of vascular plants found at the small gully catchment area belong to 13 families and 11 orders (Table 1, Fig. 5).

**Fig. 6:**
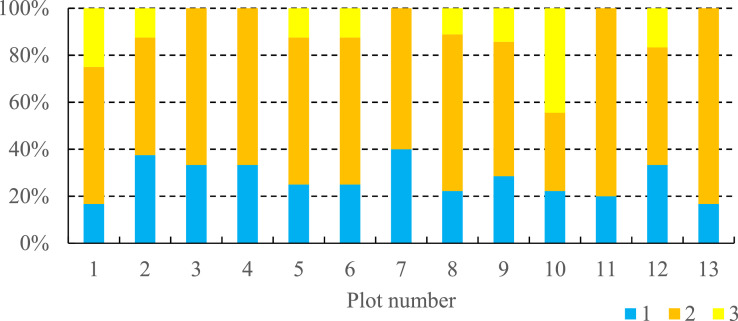
Biomorphotype of plant species occurred at the plots: 1 – dwarf semishrubs, 2 – perennials, 3 – annuals.

Most species are perennials (Table 2, Fig. 6). The dominant species are represented by dwarf semishrubs (*Artemisia lerchiana* and just in Solonetz *A. pauciflora*) and perennials (*A. austriaca, Festuca valesiaca*, and *Poa bulbosa*).

**Table 2:** Phytocoenotic characteristics of the plant communities occurred in the gully catchment area located at the NE Ergeni Upland.

	Key plot number and soils: K – Kastanozems, Sn – Solonetz												
	1	2	3	4	5	6	7	8	9	10	11	12	13
Species [3]	K	Sn	K	K	K	K	K	K	Sn	K	K	Sn	K
**Dwarf semishrubs**													
*Artemisia lerchiana*	sp.	cop.1gr	cop.1	–	sp.	sp.	sp.	cop.1	cop.1	sol.	–	cop.2	–
*Artemisia pauciflora*	–	cop.2	–	–	–	–	–	–	–	–	–	cop.2	–
*Artemisia santonicа*	sp.	–	–	–	–	–	–	–	–	–	–	–	–
**Perennials**													
*Agropyron desertorum*	–	–	–	–	–	–	–	–	–	–	–	–	sp.
*Allium* sp.	sp.gr	–	–	–	–	–	–	–	–	–	–	–	–
*Artemisia austriaca*	–	cop.1	cop.2	cop.2	cop.2	cop.2	cop.1-2	cop.2	cop.2	cop.2	cop.1	–	cop.1
*Astragalus* sp.	–	–	–	–	–	sol.	–	–	–	–	–	–	–
*Carex supina*	–	–	sp.	–	–	–	–	sp.	–	–	–	–	–
*Festuca valesiaca*	cop.2	cop.2	cop.2	cop.2	cop.2	cop.2	cop.2	cop.2	cop.2	cop.2	cop.2	sp.	sp.
*Gagea bulbifera*	sp.	cop.2	sp.	sp.	sp.	sp.	–	–	sp.	sp.	sp.	sp.	sp.
*Geranium* sp.	sp.gr	–	–	–	–	–	–	–	sol.	–	sp.	–	–
*Myosotis* sp.	cop.2	–	–	–	–	sp.	–	sp.	–	–	–	sp.	–
*Poa bulbosa*	cop.3	cop.3	cop.2	–	cop.1	cop.2	cop.2	cop.2	–	cop.2	cop.1	cop.3	cop.2
*Stipa capillata*	–	–	–	–	–	–	–	–	sp.gr	–	–	–	–
*Stipa lessingiana*	–	–	–	–	sp.	–	–	sol.	–	–	–	–	–
*Stipa sareptana*	–	–	–	–	sp.	–	–	sp.	–	–	–	–	–
*Tanacetum achilleifolium*	sp.	cop.2	–	–	–	–	sol.	–	–	–	–	–	–
**Annuals**													
*Cerastium* sp.	sp.	–	–	–	sp.	cop.1	–	sp.	sp.	sp.	–	cop.1	–
*Ceratocephala testiculata*	sp.gr	sp.	–	–	–	–	–	–	–	–	–	–	–
*Erophila verna*	cop.2	–	–	–	–	–	–	–	–	–	–	–	–
*Lappula squarrosa*	–	–	–	–	–	–	–	–	–	sol.	–	–	–
*Petrosimonia triandra*	–	–	–	–	–	–	–	–	–	sol.	–	–	–
*Polygonum patulum*	–	–	–	–	–	–	–	–	–	sol.	–	–	–

Abundance of species according to the Drude method: *Copiosae* (cop.3 – very abundant, cop.2 – abundant, there are many individuals of this species, cop.1 – abundant), sp. – *Sparsae* (plants are found occasionally, scattered, in small numbers), sol. – *Solitariae* (plants are rare or single); gr – in groups.

**Fig. 7:**
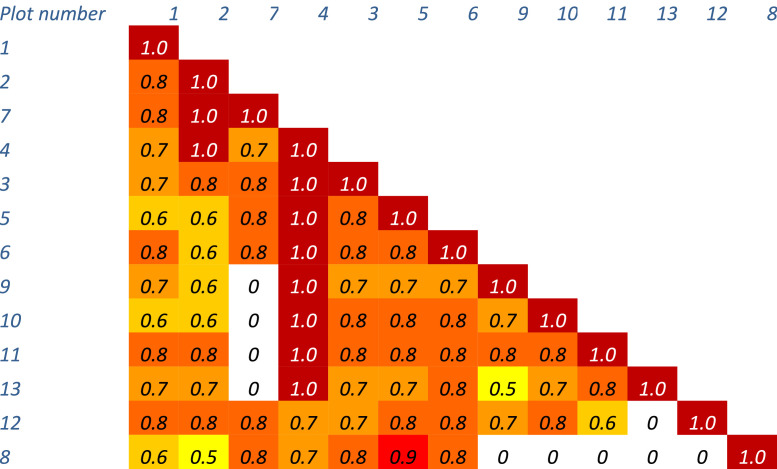
Matrix of the similarity of the species composition of plant communities described at the plots 1 – 13.

**Fig. 8:**
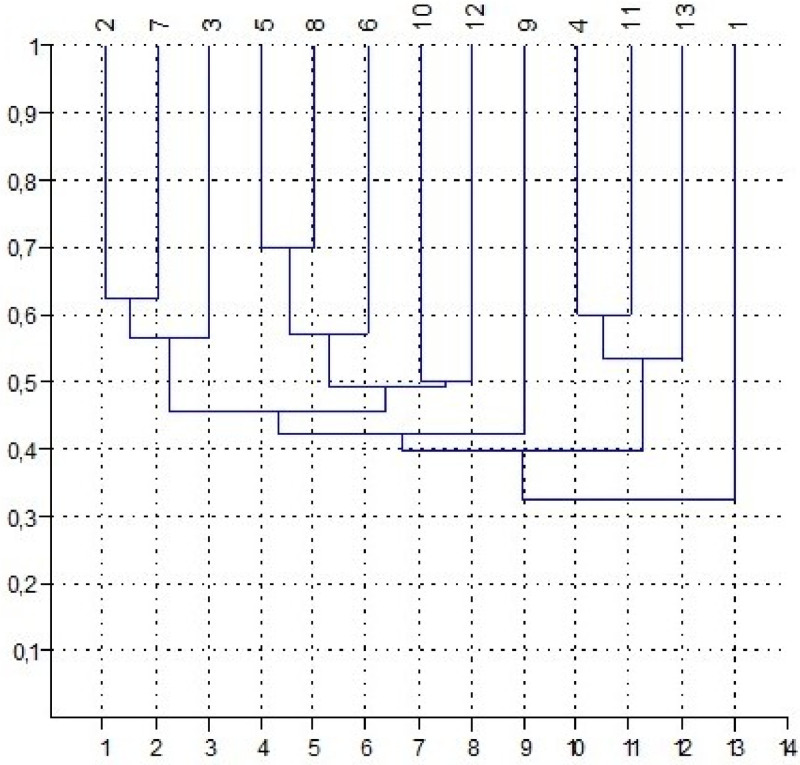
Dendrogram of the similarity based on the Jacquard coefficient of the species composition of communities located at the NE Ergeni Upland.

**Fig. 9:**
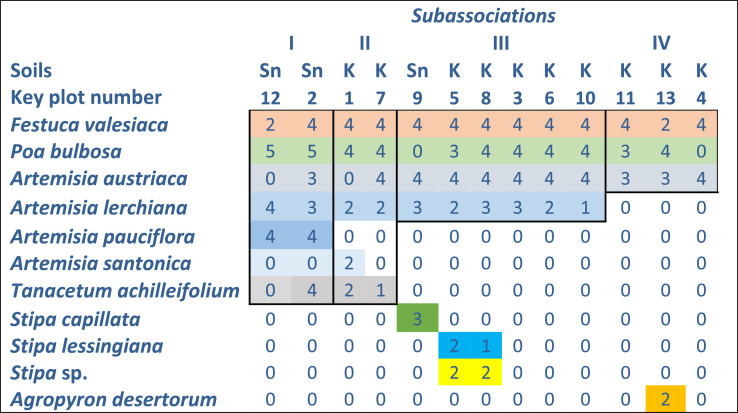
Plant communities, species dominants and indicators. Soils: K – Kastanozems, Sn – Solonetz. Abundance of plant species based on the Drude method: 1 – sol; 2–sp.; 3 – cop1; 4 – cop2 – 4; 5 – cop3.

As the similarity of communities is more than 30% (Fig. 7) based on the Jacquard coefficient (the species composition of communities, the phytocenotic role of dominants and their indicative value in relation to soil are not considered), they attribute to the same association and 4 subassociations (Figs. 8; 9): I – *Artemisia pauciflora*+*Poa bulbosa* (with *Artemisia lerchiana, Festuca valesiaca, Tanacetum achilleifolium*); II – *Festuca valesiaca*+*Poa bulbosa* (with *Tanacetum achilleifolium, Artemisia lerchiana*); III – *Festuca valesiaca*+*Artemisia austriaca*+*Poa bulbosa* +*Artemisia lerchiana*; IV – *Festuca valesiaca* +*Artemisia austriaca*+*Poa bulbosa*.

**Fig. 10:**
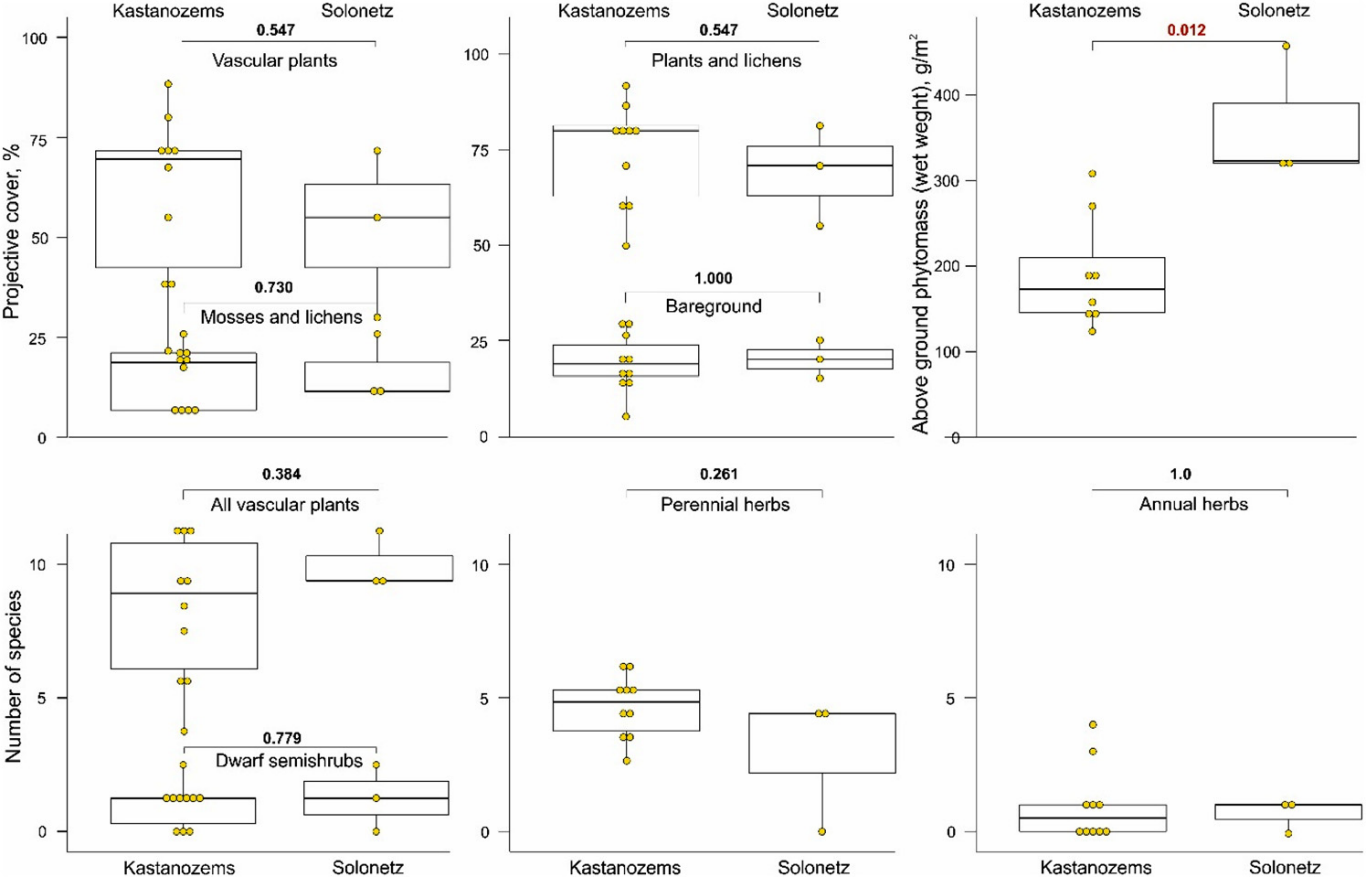
Selected characteristics of plant cover grown on Kastanozems and Solonetz studied at the small gully catchment area occurred at the NE Ergeni Upland.

Kastanozems and Solonetz are forming under dwarf semishrubs and herbs (Table 3). At Solonetz, it is higher projective cover of vascular plants and above ground phytomass (Fig. 10).

**Table 3:** Selected features of the plant communities at the small gully catchment area occurred at the NE Ergeni Upland.

				Projective cover, %					Number of species			
Key plot	Plant community	Soils	Loca-tion	Vascular plants	Bareground	Mosses and lichens	Total (plants and lichens)	Above ground Phytomass (wet weight), g/m^2^	Dwarf semishrubs	Perennials	Annuals	Total
1	*Festuca valesiaca+Poa bulbosa*	K	I	70	15	15	85	308	2	7	3	12
2	*Artemisia pauciflora+Poa bulbosa*	Sn	I	70	20	25	80	457	2	5	1	8
3	*Festuca valesiaca+Artemisia austriaca+Poa bilbosa +Artemisia lerchiana*	K	I	70	15	15	85	188	1	5	0	6
4	*Festuca valesiaca +Artemisia austriaca+Poa bulbosa*	K	Gb	60	25	15	75	142	0	3	0	3
5	*Festuca valesiaca+Artemisia austriaca+Poa bilbosa +Artemisia lerchiana*	K	Gb	60	25–30	15–20	75	147	1	6	1	8
6	*Festuca valesiaca+Artemisia austriaca+Poa bilbosa +Artemisia lerchiana*	K	Gb	70	20	20	80	NA	1	6	1	8
7	*Festuca valesiaca+ Poa bulbosa*	K	Gb	75	20	15–20	80	190	1	4	0	5
8	*Festuca valesiaca+Artemisia austriaca+Poa bilbosa +Artemisia lerchiana*	K	Gb	75	15–20	5	80	158	1	7	1	9
9	*Festuca valesiaca+Artemisia austriaca+Poa bilbosa +Artemisia lerchiana*	Sn	Gb	70	25	10	75	323	1	5	1	7
10	*Festuca valesiaca+Artemisia austriaca+Poa bilbosa +Artemisia lerchiana*	K	Gb	75	5	20	95	NA	1	4	4	9
11	*Festuca valesiaca +Artemisia austriaca+Poa bulbosa*	K	Gb	75	15–20	25	80	124	0	5	0	5
12	*Artemisia pauciflora+Poa bulbosa*	Sn	I	75	15	10	85	317	2	3	1	6
13	*Festuca valesiaca +Artemisia austriaca+Poa bulbosa*	K	I	65	25–30	5	70	270	0	6	0	6

K – Kastanozems, Sn – Solonetz, I – interfluve, Gb – gully bottom, NA – not measured.

## 2 Experimental Design, Materials and Methods

**Fig. 11:**
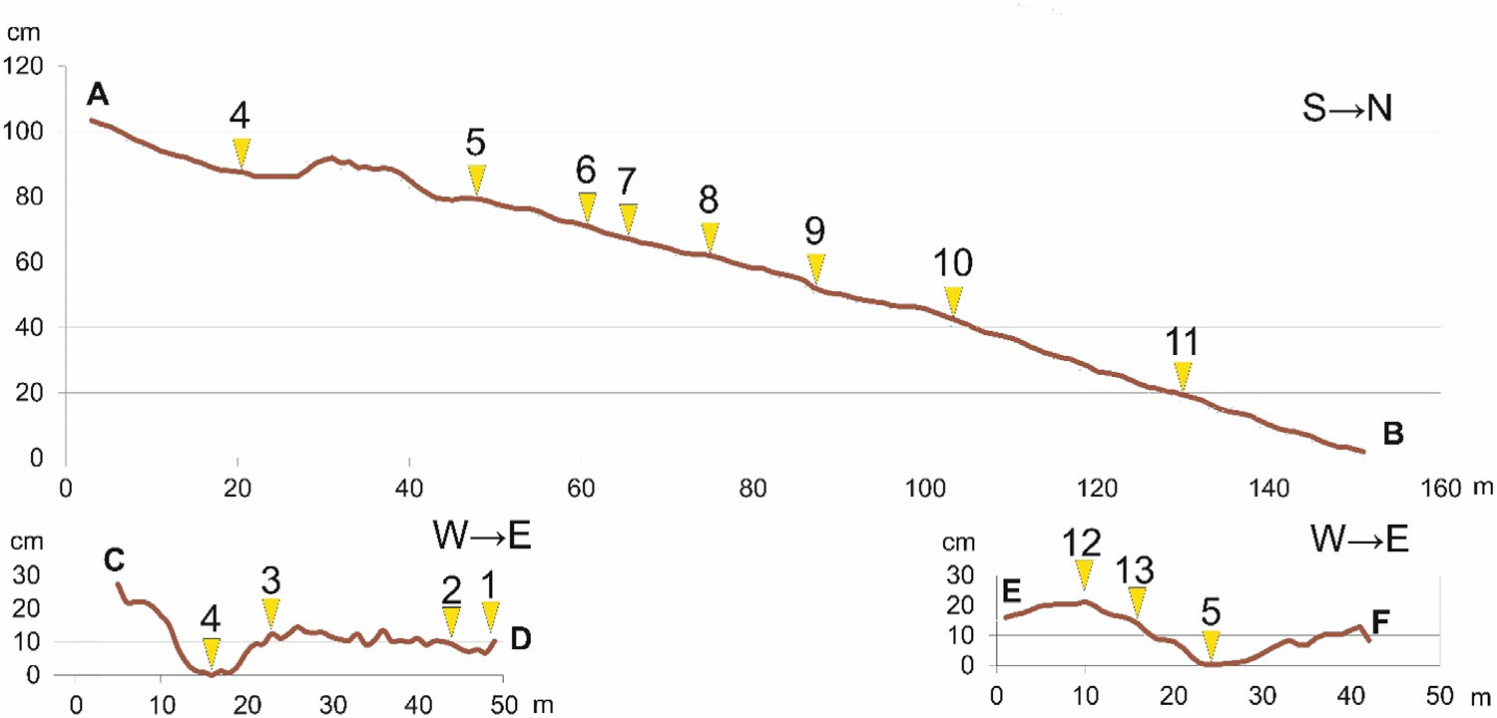
Leveling survey was carried out along three profiles. Numbers – key plots, letters (A – F) – the first and last points of leveling survey (see Fig. 1).

Leveling survey was carried out along three profiles (Fig. 11).

Phylogenetic tree of species found at the gully catchment area was built on a basis of a phylogenetic tree using scripts and instructions from [2]. Two species absent in an initial tree [4] (*Artemisia lerchiana* and *Petrosimonia triandra*) named according to [3]) were added to basal nodes of their families using scripts provided in [4].

A total of 79 soil samples (500–700 g each) were collected from a depth 0 – 130 cm (A, B and C soil horizons of Kastanozems and Solonetz). Plastic and steel tools were used for sampling. After air-drying and declumping the aggregates, the soil was sieved through a 1 mm mesh sieve. In soil samples, particle-size distribution, elemental composition (total concentration of 26 chemical elements), and total organic carbon content (dichromate digestion based on Walkley-Black method) were measured. The particle-size distribution was analyzed using a laser diffraction technique and an ‘Analysette 22’ equipment (Germany) in samples pre-treated with 4% Na_4_P_2_O_7_. CaCO_3_ concentration was analyzed by a manometric measurement of the CO_2_ released following acid (HCl) dissolution [8].The total content of chemical elements was measured using an X-Ray fluorescence technique and a PANalytical spectrometer (Netherlands) as described in details in [9,10].

A soil water extracts were prepared to measure pH value (1:2.5 soil:water ratio) and electrical conductivity (a 1:5 soil:water ratio).

Mobile fractions (F1–F3) were obtained according to the extraction procedure by [11] with the use of the following reagents: F1 (ChE1) – with NH_4_Ac (ammonium acetate buffer) and the soil:solution ratio of 1:5, F2 (ChE2) – with 1% EDTA (ethylenediaminetetraacetic acid) in NH_4_Ac and the soil:solution ratio of 1:5 and F3 (ChE3) – with 1M HNO_3_ and the soil:solution ratio of a 1:10. Concentrations of the extracted ChEs in the filtrates were determined using an ‘iCAP-6500’ (Inductively Coupled Plasma Atomic Emission Spectrometer by ‘Thermo Scientific’, USA).

**Table 4:** Mineralogical composition of Kastanozems and Solonetz studied at the gully catchment area (NE Ergeni Upland).

Soil	Horizon	Smectite	Illite	I/Sm	Kl	Chlorite	Pl	FS	Q	Calcite	D	G
Kastanozems	A	11	**8,8**	**15**	2,0	0,9	**13**	4,1	**44**	1,1	<0,5	<0,5
	B	**18**	7,0	10	**3,4**	0,9	8	**4,7**	40	6,9	1,7	<0,5
	Bk	13	7,2	12	3,2	1,5	11	4,4	38	**8,4**	1,7	<0,5
	Cy	12	7,1	14	2,5	**2,2**	9	2,9	36	5,9	**3,3**	**4,8**

Solonetz	E	1	6,3	13	1,7	1,0	**16**	**6,6**	**55**	0,6	<0,5	<0,5
	Bn	**21**	**7,8**	14	**2,7**	1,0	10	5,7	35	1,5	0,6	<0,5
	Bk	9	5,8	**19**	2,3	**3,1**	12	4,4	32	**11,3**	2,0	<0,5
	Cz	8	6,1	**19**	2,5	0,9	12	4,4	40	4,8	**2,1**	<0,5

D – Dolomite, G – Gypsum, I/Sm – illite-smectite mixed-layer minerals with predomination of illite interlayers, Kl – Kaolinite, Pl – Plagioclases, FS – feldspars, Q – Quartz. Maximal concentration of the mineral phase are marked in bold separetely for Kastanozems and Solonetz.

**Table 5:** Correlation matrix for soil proxies (all data set for Kastanozems and Solonetz studied at the gully catchment area).

Proxy	500-250	250-50	50-10	10-5	5-1	<1	EC	pH	TOC	LOI	Carbonates
500-250	1										
250-50	***0.367***	1									
50-10	-0.204	0.187	1								
10-5	-0.086	***-0.589***	***-0.356***	1							
5-1	***-0.242***	***-0.813***	***-0.637***	***0.609***	1						
<1	***-0.310***	***-0.738***	***-0.540***	***0.296***	***0.853***	1					
EC	-0.053	***-0.402***	***-0.377***	***0.414***	***0.435***	***0.476***	1				
pH	-0.180	***-0.515***	***-0.226***	***0.322***	***0.441***	***0.575***	***0.635***	1			
TOC	0.215	***0.471***	0.122	-0.152	***-0.359***	***-0.595***	***-0.505***	***-0.663***	1		
LOI	***-0.280***	***-0.638***	***-0.244***	0.204	***0.569***	***0.760***	***0.474***	***0.487***	***-0.495***	1	
Carbonates	***0,247***	-0,084	-0,194	0,194	0,184	0,124	0,194	0,097	-0,040	0,104	***1,000***
Na	***0.338***	***0.259***	-0.146	0.175	-0.194	***-0.309***	***0.322***	0.140	0.080	***-0.467***	***-0,490***
Mg	-0.205	***-0.583***	-0.006	***0.276***	***0.434***	***0.549***	***0.588***	***0.718***	***-0.773***	***0.451***	***0,484***
Al	-0.178	0.061	***0.226***	0.116	-0.026	***-0.240***	-0.201	-0.186	0.107	***-0.481***	***-0,483***
Si	***0.306***	***0.723***	***0.270***	***-0.314***	***-0.652***	***-0.814***	***-0.557***	***-0.557***	***0.601***	***-0.960***	***-0,938***
K	0.070	***0.408***	***0.343***	-0.122	***-0.407***	***-0.624***	***-0.408***	***-0.438***	***0.432***	***-0.774***	***-0,821***
Ca	***-0.315***	***-0.668***	***-0.227***	***0.226***	***0.585***	***0.788***	***0.495***	***0.559***	***-0.613***	***0.949***	***0,965***
Ti	0.113	***0.380***	***0.381***	-0.076	***-0.398***	***-0.628***	***-0.493***	***-0.475***	***0.470***	***-0.766***	***-0,761***
Mn	***0.284***	***0.633***	***0.350***	***-0.322***	***-0.609***	***-0.779***	***-0.547***	***-0.582***	***0.624***	***-0.873***	***-0,906***
Fe	-0.218	-0.138	0.162	***0.232***	0.152	-0.055	-0.117	-0.087	0.010	***-0.277***	***-0,223***
P	-0.175	***-0.316***	-0.078	0.129	***0.254***	***0.439***	***0.379***	***0.348***	***-0.332***	***0.679***	***0,567***
S	0.170	-0.041	***-0.307***	0.218	0.142	0.067	***0.512***	0.076	0.015	0.160	0,021
Cr	0.056	***0.506***	0.138	-0.146	***-0.402***	***-0.593***	***-0.399***	***-0.493***	***0.455***	***-0.773***	***-0,791***
V	-0.053	0.150	0.177	0.106	-0.088	***-0.363***	***-0.285***	***-0.289***	***0.324***	***-0.504***	***-0,495***
Co	0.092	0.155	0.135	-0.042	-0.180	***-0.334***	-0.178	***-0.221***	***0.264***	***-0.379***	***-0,408***
Ni	***-0.303***	***-0.499***	-0.084	***0.423***	***0.525***	***0.320***	0.086	0.051	-0.169	0.169	***0,417***
Cu	-0.161	0.007	0.034	0.114	0.125	-0.065	-0.121	***-0.296***	***0.308***	-0.169	-0,134
Zn	0.076	***0.325***	***0.312***	-0.141	***-0.289***	***-0.525***	***-0.360***	***-0.482***	***0.548***	***-0.569***	***-0,717***
Rb	0.037	***0.270***	***0.317***	-0.026	***-0.248***	***-0.497***	***-0.314***	***-0.361***	***0.385***	***-0.685***	***-0,760***
Sr	***-0.251***	***-0.583***	-0.192	0.179	***0.464***	***0.695***	***0.577***	***0.655***	***-0.717***	***0.820***	***0,825***
Zr	***0.312***	***0.741***	***0.223***	***-0.303***	***-0.644***	***-0.809***	***-0.553***	***-0.565***	***0.598***	***-0.943***	***-0,955***
Ba	***0.292***	***0.617***	***0.316***	***-0.266***	***-0.584***	***-0.736***	***-0.440***	***-0.494***	***0.493***	***-0.861***	***-0,860***
Th	***0.220***	0.019	0.014	0.061	0.005	-0.192	-0.110	-0.075	0.154	***-0.281***	***-0,271***
Y	0.016	***0.305***	***0.220***	-0.020	***-0.221***	***-0.462***	***-0.381***	***-0.337***	***0.421***	***-0.634***	***-0,648***
Nb	***0.262***	***0.702***	***0.319***	***-0.339***	***-0.657***	***-0.785***	***-0.578***	***-0.594***	***0.607***	***-0.854***	***-0,887***
Pb	0.174	***0.482***	0.207	-0.176	***-0.395***	***-0.602***	***-0.370***	***-0.451***	***0.559***	***-0.706***	***-0,706***
Cl	-0.065	***-0.325***	***-0.353***	***0.487***	***0.430***	***0.326***	***0.580***	***0.461***	-0.185	***0.272***	***0,300***
Li1	***-0.233***	***-0.688***	***-0.270***	***0.332***	***0.584***	***0.770***	***0.623***	***0.682***	***-0.706***	***0.802***	***0,807***
Be1	***-0.245***	***-0.518***	-0.143	0.125	***0.385***	***0.679***	***0.480***	***0.603***	***-0.754***	***0.797***	***0,772***
B1	-0.158	***-0.445***	***-0.253***	***0.332***	***0.377***	***0.493***	***0.854***	***0.705***	***-0.701***	***0.472***	***0,421***
Na1	-0.063	***-0.569***	***-0.412***	***0.627***	***0.568***	***0.549***	***0.790***	***0.706***	***-0.600***	***0.420***	***0,537***
Mg1	-0.189	***-0.702***	-0.174	***0.338***	***0.568***	***0.713***	***0.680***	***0.761***	***-0.806***	***0.707***	***0,670***
Al1	***-0.361***	***-0.454***	0.134	0.100	***0.343***	***0.429***	0.066	***0.253***	***-0.499***	***0.380***	***0,498***
Si1	***-0.320***	-0.163	***0.254***	0.069	0.094	0.066	-0.009	0.037	-0.123	0.098	0,126
P1	-0.127	***-0.276***	-0.085	0.054	0.157	***0.451***	***0.576***	***0.616***	***-0.738***	***0.540***	***0,405***
S1	-0.121	***-0.511***	-0.207	***0.274***	***0.405***	***0.605***	***0.771***	***0.660***	***-0.796***	***0.666***	***0,617***
K1	0.201	***0.454***	***0.359***	***-0.248***	***-0.480***	***-0.677***	***-0.376***	***-0.530***	***0.544***	***-0.697***	***-0,787***
Ca1	***-0.331***	***-0.692***	***-0.228***	***0.261***	***0.607***	***0.801***	***0.469***	***0.553***	***-0.618***	***0.933***	***0,970***
Ti1	***-0.249***	***-0.626***	-0.216	0.183	***0.531***	***0.774***	***0.453***	***0.528***	***-0.609***	***0.937***	***0,978***
V1	***-0.304***	***-0.427***	-0.123	0.075	***0.341***	***0.639***	***0.357***	***0.490***	***-0.594***	***0.819***	***0,794***
Cr1	***-0.244***	***-0.702***	-0.200	***0.229***	***0.570***	***0.775***	***0.489***	***0.616***	***-0.689***	***0.861***	***0,893***
Mn1	0.156	-0.209	0.219	-0.197	-0.020	0.161	-0.103	-0.027	***-0.303***	***0.279***	0,168
Fe1	-0.145	***-0.519***	0.063	-0.065	***0.298***	***0.544***	0.143	***0.393***	***-0.651***	***0.577***	***0,608***
Co1	0.138	***-0.326***	-0.101	0.124	***0.223***	***0.280***	0.102	0.043	-0.201	***0.336***	***0,323***
Ni1	0.217	0.120	-0.016	0.132	0.002	***-0.294***	***-0.358***	***-0.434***	***0.568***	***-0.342***	***-0,276***
Cu1	-0.172	***-0.443***	0.055	-0.024	***0.239***	***0.545***	***0.390***	***0.514***	***-0.865***	***0.572***	***0,470***
Zn1	***0.267***	0.162	0.005	***-0.254***	***-0.239***	-0.092	0.056	-0.074	0.172	0.045	-0,194
Sr1	***-0.318***	***-0.687***	-0.181	***0.232***	***0.557***	***0.758***	***0.523***	***0.651***	***-0.690***	***0.878***	***0,915***
Mo1	***-0.245***	***-0.518***	-0.143	0.125	***0.385***	***0.679***	***0.480***	***0.603***	***-0.754***	***0.797***	***0,772***
Ba1	***-0.316***	-0.153	***0.343***	***-0.350***	0.007	***0.226***	***-0.352***	-0.010	***-0.242***	***0.312***	***0,339***
Cd1	***0.356***	-0.001	0.052	***-0.238***	-0.135	0.070	0.040	0.018	0.043	***0.314***	***0,246***
Pb1	***-0.305***	***-0.618***	-0.145	0.119	***0.493***	***0.749***	***0.426***	***0.530***	***-0.674***	***0.857***	***0,864***
Li2	***-0.275***	***-0.349***	-0.129	-0.005	***0.349***	***0.547***	***0.250***	***0.288***	***-0.431***	***0.526***	***0,598***
Be2	***-0.278***	***-0.337***	-0.030	-0.028	0.214	***0.536***	***0.404***	***0.504***	***-0.770***	***0.636***	***0,579***
B2	-0.064	-0.189	***-0.226***	***0.233***	***0.234***	0.176	***0.289***	***0.308***	-0.051	0.215	0,161
Na2	-0.065	0.076	0.054	-0.003	-0.114	-0.177	-0.007	-0.035	0.146	-0.180	-0,158
Mg2	-0.062	-0.109	***-0.230***	0.164	***0.227***	0.119	0.071	-0.020	0.165	0.112	0,153
Al2	0.052	***0.381***	0.201	-0.120	***-0.256***	***-0.516***	***-0.522***	***-0.555***	***0.711***	***-0.576***	***-0,625***
Si2	***-0.300***	-0.212	-0.042	0.174	***0.293***	0.215	0.022	0.036	-0.074	0.088	0,162
P2	-0.140	-0.161	0.058	-0.056	0.010	***0.317***	***0.434***	***0.558***	***-0.762***	***0.342***	***0,225***
S2	-0.074	***-0.454***	-0.209	0.218	***0.349***	***0.551***	***0.666***	***0.640***	***-0.695***	***0.646***	***0,636***
K2	0.096	***0.349***	0.174	***-0.247***	***-0.387***	***-0.451***	***-0.243***	***-0.285***	***0.390***	***-0.369***	***-0,442***
Ca2	-0.209	***-0.259***	-0.092	0.063	0.215	***0.330***	0.165	***0.242***	***-0.303***	***0.455***	***0,480***
Ti2	0.112	-0.163	0.022	-0.100	0.029	0.210	0.072	0.111	-0.064	***0.432***	***0,501***
V2	***-0.261***	0.055	-0.013	0.166	0.087	-0.130	-0.101	-0.170	***0.228***	***-0.234***	-0,205
Cr2	***0.252***	***0.371***	0.169	-0.122	***-0.351***	***-0.545***	***-0.425***	***-0.477***	***0.642***	***-0.555***	***-0,600***
Mn2	***0.246***	***0.723***	***0.295***	***-0.343***	***-0.654***	***-0.807***	***-0.521***	***-0.587***	***0.680***	***-0.872***	***-0,937***
Fe2	***0.316***	***0.388***	***0.301***	-0.185	***-0.415***	***-0.623***	***-0.461***	***-0.484***	***0.531***	***-0.732***	***-0,686***
Co2	0.216	***0.698***	***0.292***	***-0.342***	***-0.607***	***-0.766***	***-0.594***	***-0.651***	***0.740***	***-0.822***	***-0,876***
Ni2	0.161	***0.626***	***0.247***	***-0.240***	***-0.531***	***-0.728***	***-0.467***	***-0.557***	***0.695***	***-0.819***	***-0,901***
Cu2	0.188	***0.485***	0.217	-0.120	***-0.410***	***-0.654***	***-0.489***	***-0.628***	***0.874***	***-0.589***	***-0,580***
Zn2	***0.345***	***0.438***	0.079	***-0.272***	***-0.440***	***-0.397***	-0.027	-0.008	***0.225***	***-0.296***	***-0,415***
Sr2	-0.147	***-0.253***	-0.219	***0.308***	***0.254***	***0.260***	***0.450***	***0.403***	***-0.321***	***0.269***	***0,281***
Mo2	***-0.278***	***-0.337***	-0.030	-0.028	0.214	***0.536***	***0.404***	***0.504***	***-0.770***	***0.636***	***0,579***
Ba2	-0.117	0.002	0.204	***-0.365***	-0.160	0.185	-0.066	***0.223***	***-0.252***	***0.371***	***0,374***
Cd2	***0.302***	***0.529***	0.077	-0.197	***-0.382***	***-0.579***	***-0.439***	***-0.540***	***0.743***	***-0.589***	***-0,523***
Pb2	***0.253***	***0.575***	***0.337***	***-0.317***	***-0.620***	***-0.699***	***-0.269***	***-0.353***	***0.447***	***-0.666***	***-0,789***
Li3	-0.199	***-0.499***	***-0.280***	***0.514***	***0.531***	***0.446***	***0.573***	***0.532***	***-0.603***	0.211	***0,317***
Be3	0.148	***0.418***	0.139	-0.014	***-0.281***	***-0.611***	***-0.465***	***-0.593***	***0.703***	***-0.718***	***-0,756***
B3	-0.122	***-0.312***	***-0.237***	***0.234***	***0.265***	***0.412***	***0.770***	***0.660***	***-0.571***	***0.447***	***0,434***
Na3	0.090	-0.007	0.026	-0.072	-0.042	0.009	0.161	***0.271***	0.033	0.052	-0,013
Mg3	***-0.321***	***-0.684***	-0.136	0.201	***0.564***	***0.738***	***0.490***	***0.597***	***-0.772***	***0.785***	***0,775***
Al3	-0.100	0.151	0.033	0.101	0.031	***-0.246***	***-0.304***	***-0.457***	***0.604***	***-0.295***	***-0,342***
Si3	-0.070	0.001	-0.126	0.142	0.167	0.018	-0.083	-0.122	0.081	-0.148	-0,172
P3	-0.182	***-0.415***	-0.007	0.079	***0.253***	***0.517***	***0.518***	***0.608***	***-0.845***	***0.548***	***0,432***
S3	-0.084	***-0.470***	***-0.240***	***0.283***	***0.384***	***0.543***	***0.736***	***0.611***	***-0.717***	***0.612***	***0,618***
K3	0.076	0.156	0.134	-0.097	-0.187	-0.177	0.208	0.053	-0.087	***-0.246***	***-0,405***
Ca3	***-0.253***	***-0.490***	-0.143	0.043	***0.414***	***0.626***	***0.352***	***0.427***	***-0.655***	***0.734***	***0,757***
Ti3	0.111	-0.163	0.036	-0.077	0.024	0.218	0.173	***0.222***	***-0.558***	0.186	0,119
V3	***-0.253***	***-0.254***	***-0.297***	***0.367***	***0.452***	***0.232***	0.132	-0.015	0.135	0.155	***0,319***
Cr3	-0.080	0.016	-0.099	***0.333***	0.133	-0.138	0.081	-0.045	0.083	***-0.405***	***-0,374***
Mn3	***0.320***	***0.693***	***0.235***	***-0.221***	***-0.606***	***-0.794***	***-0.410***	***-0.572***	***0.747***	***-0.782***	***-0,853***
Fe3	0.061	-0.115	0.030	0.202	0.113	-0.075	0.064	0.012	-0.142	***-0.309***	***-0,324***
Co3	***0.232***	***0.476***	0.116	-0.142	***-0.359***	***-0.593***	***-0.473***	***-0.641***	***0.935***	***-0.526***	***-0,475***
Ni3	0.168	***0.466***	0.083	-0.051	***-0.292***	***-0.578***	***-0.401***	***-0.572***	***0.802***	***-0.652***	***-0,685***
Cu3	***0.249***	***0.575***	***0.246***	-0.149	***-0.473***	***-0.723***	***-0.417***	***-0.602***	***0.747***	***-0.746***	***-0,827***
Zn3	***0.305***	***0.606***	0.214	***-0.260***	***-0.512***	***-0.713***	***-0.448***	***-0.548***	***0.618***	***-0.798***	***-0,864***
Sr3	-0.075	***-0.377***	***-0.349***	***0.253***	***0.430***	***0.501***	***0.579***	***0.508***	***-0.527***	***0.516***	***0,516***
Mo3	***0.235***	***0.509***	0.174	-0.119	***-0.396***	***-0.685***	***-0.492***	***-0.615***	***0.747***	***-0.775***	***-0,754***
Ba3	***0.356***	***0.282***	-0.073	0.183	-0.170	***-0.388***	0.008	-0.194	***0.417***	***-0.530***	***-0,557***
Cd3	***0.326***	***0.631***	0.172	***-0.308***	***-0.531***	***-0.717***	***-0.409***	***-0.513***	***0.628***	***-0.759***	***-0,846***
Pb3	***0.305***	***0.511***	***0.276***	-0.188	***-0.457***	***-0.701***	***-0.406***	***-0.493***	***0.653***	***-0.751***	***-0,824***
Na4	***0.359***	***0.318***	-0.118	0.113	***-0.253***	***-0.344***	***0.282***	0.097	0.105	***-0.498***	***-0,535***
Mg4	***-0.251***	-0.075	0.066	***0.225***	0.124	-0.003	0.033	0.083	-0.123	-0.186	-0,164
Al4	-0.174	0.044	***0.235***	0.121	-0.025	***-0.236***	-0.183	-0.158	0.063	***-0.487***	***-0,471***
Si4	***0.306***	***0.723***	***0.271***	***-0.314***	***-0.653***	***-0.815***	***-0.556***	***-0.555***	***0.601***	***-0.960***	***-0,937***
K4	0.004	***0.350***	***0.357***	-0.075	***-0.352***	***-0.570***	***-0.407***	***-0.394***	***0.363***	***-0.751***	***-0,778***
Ca4	0.148	***0.368***	0.146	-0.015	***-0.307***	***-0.511***	***-0.226***	***-0.264***	***0.488***	***-0.530***	***-0,639***
Ti4	0.123	***0.408***	***0.357***	-0.073	***-0.406***	***-0.647***	***-0.509***	***-0.486***	***0.517***	***-0.788***	***-0,790***
Mn4	***-0.232***	***-0.662***	***-0.239***	***0.237***	***0.588***	***0.763***	***0.405***	***0.541***	***-0.726***	***0.747***	***0,839***
Fe4	***-0.230***	-0.141	0.157	***0.234***	0.163	-0.041	-0.133	-0.096	0.026	***-0.251***	-0,184
P4	0.133	***0.335***	0.029	-0.081	-0.193	***-0.419***	***-0.496***	***-0.613***	***0.881***	***-0.301***	***-0,295***
S4	***0.240***	***0.247***	***-0.256***	0.131	-0.029	***-0.269***	0.155	***-0.325***	***0.468***	***-0.245***	***-0,352***
Cr4	0.063	***0.513***	0.142	-0.161	***-0.415***	***-0.597***	***-0.403***	***-0.495***	***0.454***	***-0.769***	***-0,787***
V4	0.002	0.176	***0.221***	0.065	-0.144	***-0.420***	***-0.343***	***-0.307***	***0.316***	***-0.579***	***-0,551***
Co4	-0.146	***-0.365***	-0.023	0.135	***0.258***	***0.337***	***0.290***	***0.363***	***-0.650***	***0.247***	***0,261***
Ni4	***-0.292***	***-0.665***	-0.058	***0.251***	***0.534***	***0.618***	***0.312***	***0.332***	***-0.590***	***0.645***	***0,681***
Cu4	***-0.311***	***-0.524***	-0.159	0.165	***0.500***	***0.685***	***0.386***	***0.400***	***-0.591***	***0.630***	***0,654***
Zn4	-0.090	0.142	***0.289***	-0.015	-0.114	***-0.347***	***-0.289***	***-0.382***	***0.397***	***-0.430***	***-0,537***
Sr4	***0.272***	***0.615***	***0.271***	***-0.249***	***-0.570***	***-0.731***	***-0.490***	***-0.531***	***0.614***	***-0.820***	***-0,887***
Ba4	***0.326***	***0.603***	0.156	-0.165	***-0.508***	***-0.699***	***-0.308***	***-0.467***	***0.460***	***-0.859***	***-0,875***
Pb4	0.043	***0.346***	0.130	-0.042	***-0.237***	***-0.439***	***-0.274***	***-0.312***	***0.418***	***-0.597***	***-0,522***
Na_m	0.075	***-0.227***	***-0.277***	***0.464***	***0.263***	0.133	***0.623***	***0.512***	-0.172	0.063	0,141
Mg_m	***-0.294***	***-0.717***	***-0.230***	***0.277***	***0.623***	***0.784***	***0.562***	***0.653***	***-0.745***	***0.829***	***0,854***
Al_m	-0.099	0.085	-0.087	0.155	0.123	-0.165	***-0.270***	***-0.426***	***0.670***	-0.159	-0,128
Si_m	***-0.266***	***-0.387***	***-0.239***	***0.305***	***0.501***	***0.444***	0.215	0.180	-0.183	***0.387***	***0,454***
K_m	0.125	***0.408***	***0.256***	***-0.253***	***-0.431***	***-0.548***	-0.135	***-0.345***	***0.421***	***-0.549***	***-0,677***
Ca_m	***-0.276***	***-0.512***	-0.171	0.107	***0.451***	***0.664***	***0.384***	***0.437***	***-0.660***	***0.739***	***0,790***
Ti_m	-0.097	***-0.455***	-0.167	0.035	***0.337***	***0.609***	***0.413***	***0.457***	***-0.645***	***0.701***	***0,744***
Mn_m	***0.243***	***0.684***	***0.293***	***-0.278***	***-0.627***	***-0.794***	***-0.438***	***-0.550***	***0.697***	***-0.807***	***-0,901***
Fe_m	***0.393***	0.184	0.088	-0.120	***-0.260***	***-0.262***	0.013	-0.064	-0.023	***-0.324***	***-0,425***
P_m	-0.178	***-0.356***	-0.016	0.025	0.207	***0.493***	***0.508***	***0.629***	***-0.902***	***0.480***	***0,366***
S_m	-0.097	***-0.491***	-0.168	***0.233***	***0.356***	***0.567***	***0.687***	***0.642***	***-0.821***	***0.613***	***0,566***
Cr_m	-0.111	***-0.472***	***-0.289***	***0.562***	***0.562***	***0.360***	***0.344***	***0.336***	***-0.225***	0.162	***0,305***
V_m	***-0.312***	***-0.324***	***-0.323***	***0.313***	***0.477***	***0.387***	***0.321***	0.192	-0.002	***0.383***	***0,472***
Co_m	0.171	***0.491***	0.073	-0.172	***-0.350***	***-0.514***	***-0.458***	***-0.534***	***0.832***	***-0.462***	***-0,414***
Ni_m	***0.230***	***0.686***	0.173	***-0.272***	***-0.549***	***-0.726***	***-0.470***	***-0.574***	***0.764***	***-0.800***	***-0,837***
Cu_m	***0.277***	***0.604***	***0.294***	-0.173	***-0.562***	***-0.794***	***-0.477***	***-0.619***	***0.780***	***-0.746***	***-0,772***
Zn_m	***0.387***	***0.586***	0.037	***-0.255***	***-0.468***	***-0.563***	***-0.297***	***-0.382***	***0.444***	***-0.596***	***-0,726***
Sr_m	***-0.289***	***-0.634***	***-0.283***	***0.245***	***0.581***	***0.768***	***0.570***	***0.601***	***-0.674***	***0.876***	***0,886***
Ba_m	***-0.282***	***-0.267***	***0.233***	-0.187	0.112	***0.325***	-0.099	0.108	***-0.253***	***0.457***	***0,474***
Pb_m	0.164	-0.021	0.045	-0.186	-0.093	0.054	0.098	0.049	-0.066	***0.306***	0,081

Spearman correlation rank ratios with p-value< 0.05 and | r | > 0.22 are marked in bold Italic.

**Table 6:** P-values of the Mann-Whitney U-test for different data sets characterized soils situated at the NE Ergeni Upland.

		Kastanozems: interfluve and gully bottom			Kastanozems (all)			Solonetz (all)			Kastanozems and Solonetz		
Analyte		T	S	PM	T & PM	T & S	S & PM	T & PM	T & S	S & PM	T	S	PM
Particle matter diameter	1000-500	1.000	1.000	1.000	1.000	1.000	1.000	1.000	0.815	0.777	1.000	0.672	1.000
	500-250	0.903	0.659	1.000	0.041	0.058	0.803	0.881	0.484	0.336	0.906	0.840	0.170
	250-50	0.903	0.073	0.743	<0.0005	<0.0005	0.503	0.025	0.010	0.113	0.859	0.039	0.382
	50-10	0.027	0.705	0.870	0.951	0.194	0.286	0.025	0.052	0.571	0.953	0.005	0.018
	10-5	0.806	0.900	0.768	0.130	0.003	0.058	0.025	0.024	0.257	0.515	0.002	0.001
	5-1	0.462	0.284	0.341	<0.0005	<0.0005	0.340	0.025	0.010	0.089	0.859	0.007	0.236
	<1	0.327	0.431	0.450	<0.0005	<0.0005	0.266	0.025	0.010	0.213	0.859	0.077	0.349
EC		0,794	0.002	0.053	<0.0005	<0.0005	0.318	0.053	0.048	0.036	1.000	0.001	0.001
pH		0,433	0.038	0.033	<0.0005	<0.0005	0.008	0.053	0.061	0.533	0.334	0.397	0.596
TOC		0,327	0.413	0.341	<0.0005	<0.0005	<0.0005	0.025	0.030	0.004	0.515	0.917	0.105
LOI		0,903	0.801	0.115	<0.0005	<0.0005	0.510	0.025	0.009	0.527	0.015	0.578	0.236

Total content	Na	0.806	0.006	0.001	0.001	<0.0005	0.003	0.456	0.194	0.006	0.015	0.001	0.001
	Mg	0.126	0.115	0.470	<0.0005	<0.0005	0.002	0.025	0.009	0.082	0.015	0.259	0.349
	Al	0.624	0.850	0.974	0.877	0.823	0.794	0.297	0.083	0.399	0.173	0.259	0.533
	Si	0.582	0.659	0.577	<0.0005	<0.0005	0.716	0.025	0.009	0.673	0.028	0.848	0.803
	K	0.624	0.729	0.718	<0.0005	0.003	0.364	0.551	0.516	0.874	0.515	0.566	0.365
	Ca	0.903	0.705	0.158	<0.0005	<0.0005	0.633	0.025	0.009	0.752	0.139	0.385	0.261
	Ti	0.582	0.777	0.718	0.002	0.011	0.982	0.025	0.170	0.635	0.554	0.945	0.333
	Mn	0.076	0.777	0.870	<0.0005	<0.0005	0.312	0.025	0.021	0.833	0.678	0.614	0.134
	Fe	0.806	0.659	0.896	0.404	0.204	0.901	0.025	0.014	0.225	0.021	0.259	0.318
	P	0.903	0.850	0.412	0.004	0.010	0.271	0.037	0.083	0.752	0.015	0.555	0.851
	S	0.582	0.950	0.036	0.038	0.002	0.602	0.025	0.097	0.092	0.173	0.001	0.002
	Cr	1.000	0.705	0.325	<0.0005	0.009	0.691	0.025	0.083	0.792	0.173	0.651	0.574
	V	0.903	0.950	0.922	0.011	0.464	0.312	0.371	0.097	0.527	0.124	0.741	0.399
	Co	0.759	0.900	0.212	0.020	0.009	0.447	0.766	0.829	0.598	0.058	0.487	0.473
	Ni	0.540	0.101	0.224	<0.0005	<0.0005	0.052	0.025	0.009	0.010	0.086	0.042	0.236
	Cu	0.806	0.059	0.743	0.370	0.464	0.104	0.456	0.170	0.003	0.260	0.040	0.201
	Zn	0.126	0.659	0.743	<0.0005	0.004	0.617	0.456	0.083	0.114	0.236	0.089	0.190
	Rb	0.098	0.825	0.844	0.002	0.017	0.578	0.371	0.516	0.598	0.066	0.217	0.533
	Sr	0.178	0.123	0.004	<0.0005	<0.0005	0.242	0.025	0.025	0.527	0.260	0.781	0.261
	Zr	0.903	0.682	0.325	<0.0005	<0.0005	0.812	0.025	0.009	0.598	0.044	0.876	0.349
	Ba	0.076	0.659	0.341	<0.0005	<0.0005	0.307	0.025	0.021	0.833	0.066	0.651	0.662
	Th	0.327	0.413	0.793	0.745	0.550	0.928	0.551	0.516	0.958	0.515	0.627	0.755
	Y	0.624	0.925	0.870	0.002	0.034	0.666	0.456	0.613	0.399	0.139	0.339	0.827
	Nb	0.668	0.900	0.622	<0.0005	<0.0005	0.991	0.025	0.009	0.958	0.024	0.986	0.827
	Pb	0.624	0.378	1.000	<0.0005	<0.0005	0.414	0.037	1.000	0.635	0.076	0.404	0.662
	Cl	1.000	0.027	0.450	0.865	0.332	0.212	0.025	0.009	0.008	0.139	0.001	0.001

F1	Li1	0.759	0.101	0.123	<0.0005	<0.0005	0.340	0.025	0.009	0.752	0.051	0.627	0.876
	Be1	0.540	0.659	0.670	<0.0005	0.001	0.115	0.025	0.194	0.114	0.859	0.211	0.640
	B1	1.000	0.007	<0.0005	<0.0005	<0.0005	0.001	0.025	0.014	0.045	0.906	0.006	0.010
	Na1	0.327	0.101	0.014	<0.0005	<0.0005	0.011	0.025	0.061	0.035	0.066	0.001	0.001
	Mg1	0.066	0.038	0.818	<0.0005	<0.0005	0.003	0.025	0.009	0.092	0.051	0.487	0.533
	Al1	0.270	0.115	0.001	<0.0005	<0.0005	0.427	0.025	0.009	0.343	0.594	0.251	0.007
	Si1	0.327	0.314	0.061	0.005	0.002	0.188	0.101	0.021	0.073	0.038	0.466	0.013
	P1	0.391	0.023	0.002	<0.0005	0.008	0.001	0.025	0.386	0.114	0.767	0.917	0.574
	S1	0.806	0.284	0.006	<0.0005	0.001	0.001	0.025	0.061	0.035	0.953	0.314	0.002
	K1	0.624	0.571	0.039	<0.0005	<0.0005	0.838	0.456	0.386	0.833	0.086	0.754	0.533
	Ca1	0.270	0.659	0.123	<0.0005	<0.0005	0.617	0.025	0.009	0.833	0.038	0.331	0.289
	Ti1	0.540	0.850	0.071	<0.0005	<0.0005	0.699	0.025	0.194	0.833	0.859	0.348	0.303
	V1	0.713	0.592	0.200	<0.0005	<0.0005	0.184	0.025	0.083	0.916	0.374	0.079	0.007
	Cr1	0.358	0.801	0.168	<0.0005	<0.0005	0.352	0.025	0.009	0.399	0.859	0.664	0.399
	Mn1	0.037	0.450	0.450	0.228	0.560	0.001	0.881	0.248	0.073	0.678	0.089	0.151
	Fe1	0.806	0.450	0.023	<0.0005	0.008	0.004	0.655	0.386	0.958	0.038	0.175	0.009
	Co1	0.066	0.682	0.948	0.370	0.988	0.170	0.655	0.194	0.040	0.286	0.677	0.275
	Ni1	0.806	0.637	0.237	0.001	0.069	0.001	0.025	0.149	0.429	0.953	0.331	0.975
	Cu1	0.624	0.529	0.646	<0.0005	<0.0005	<0.0005	0.025	0.112	0.015	0.813	0.651	0.061
	Zn1	0.391	0.284	0.718	0.068	0.010	0.086	0.025	0.030	0.527	0.813	0.297	0.399
	Sr1	0.111	0.147	0.178	<0.0005	<0.0005	0.733	0.025	0.009	0.673	0.066	0.651	0.092
	Mo1	0.540	0.659	0.670	<0.0005	0.001	0.115	0.025	0.194	0.114	0.859	0.211	0.640
	Ba1	0.391	0.314	0.017	0.001	<0.0005	0.666	0.025	0.773	0.003	0.038	<0.0005	0.001
	Cd1	0.270	0.900	0.108	0.486	0.370	0.847	0.025	0.009	0.673	0.155	0.165	0.365
	Pb1	0.713	0.529	0.224	<0.0005	<0.0005	0.547	0.025	0.036	0.916	0.086	0.187	0.031

F2	Li2	0.713	0.413	0.793	0.001	0.015	0.838	0.297	0.386	1.000	0.260	0.404	0.382
	Be2	0.540	0.529	0.670	<0.0005	0.001	0.055	0.025	0.386	0.065	0.859	0.231	0.640
	B2	0.066	0.257	0.470	0.578	0.893	0.633	0.655	0.516	0.752	0.594	0.639	0.708
	Na2	0.540	1.000	1.000	0.734	0.731	1.000	1.000	0.829	0.792	0.859	0.689	1.000
	Mg2	0.462	0.900	0.450	0.024	0.917	0.177	0.655	0.083	0.673	0.260	0.065	0.289
	Al2	0.327	0.314	0.001	<0.0005	0.104	0.008	0.025	0.564	0.073	0.086	0.314	0.349
	Si2	0.903	0.705	0.178	0.048	0.011	0.204	0.053	0.061	0.399	0.139	0.089	0.662
	P2	0.391	0.027	0.140	<0.0005	0.011	<0.0005	0.025	0.386	0.006	0.767	0.754	0.261
	S2	0.806	0.257	0.001	<0.0005	0.002	0.013	0.180	0.043	0.461	0.260	0.281	0.092
	K2	0.806	0.614	0.033	<0.0005	0.001	0.586	0.655	0.773	0.206	0.028	0.455	0.170
	Ca2	0.270	0.115	0.922	0.003	0.622	0.220	0.655	0.112	0.343	0.038	0.348	0.236
	Ti2	0.178	0.950	0.818	0.877	0.988	0.973	0.101	0.149	0.916	0.066	0.889	0.105
	V2	0.142	1.000	0.224	0.307	0.289	0.007	0.101	0.030	0.073	0.038	0.126	1.000
	Cr2	0.066	0.257	1.000	<0.0005	<0.0005	0.318	0.297	0.348	0.527	0.953	0.159	0.492
	Mn2	0.391	1.000	0.279	<0.0005	<0.0005	0.964	0.025	0.021	0.527	0.314	0.297	0.492
	Fe2	0.027	0.850	0.200	<0.0005	<0.0005	0.173	0.025	0.009	0.833	0.594	0.702	0.236
	Co2	0.270	0.413	0.577	<0.0005	<0.0005	0.247	0.025	0.021	0.292	0.859	0.651	0.151
	Ni2	0.540	0.850	0.178	<0.0005	<0.0005	0.540	0.025	0.665	0.292	0.110	0.154	0.382
	Cu2	0.806	0.659	0.922	<0.0005	<0.0005	<0.0005	0.025	0.386	0.004	0.515	0.165	0.105
	Zn2	0.713	0.231	0.158	0.001	<0.0005	0.302	0.456	0.061	0.268	0.515	0.404	0.382
	Sr2	1.000	0.186	0.470	0.164	0.502	0.525	0.180	0.279	0.833	1.000	0.224	0.574
	Mo2	0.540	0.529	0.670	<0.0005	0.001	0.055	0.025	0.386	0.065	0.859	0.231	0.640
	Ba2	0.624	0.614	0.431	0.008	0.066	0.407	0.025	0.248	0.058	0.260	0.052	0.001
	Cd2	0.270	0.925	0.251	<0.0005	<0.0005	0.146	0.025	0.009	0.003	0.767	0.050	0.596
	Pb2	0.903	0.284	0.082	<0.0005	<0.0005	0.109	0.180	0.312	0.598	0.173	0.211	0.851
F3	Li3	0.540	0.078	<0.0005	<0.0005	<0.0005	0.013	0.025	0.009	0.140	0.139	0.002	0.029
	Be3	0.159	0.659	0.470	<0.0005	0.020	0.061	0.025	0.829	0.114	0.374	0.266	0.755
	B3	0.086	0.007	<0.0005	<0.0005	0.013	0.184	0.025	0.030	0.399	0.286	0.022	0.134
	Na3	0.086	0.059	0.071	0.914	0.929	0.991	0.456	1.000	0.429	0.953	0.876	0.435
	Mg3	0.462	1.000	0.533	<0.0005	<0.0005	0.063	0.025	0.009	0.114	0.038	0.424	0.803
	Al3	0.806	0.208	0.008	<0.0005	0.800	<0.0005	0.456	0.030	0.011	0.051	0.297	0.618
	Si3	0.462	0.257	0.974	0.536	0.709	0.329	0.180	0.030	0.073	0.110	0.076	0.708
	P3	0.462	0.529	0.053	<0.0005	<0.0005	<0.0005	0.025	0.112	0.045	0.110	0.835	0.708
	S3	0.327	0.378	0.001	<0.0005	0.001	0.063	0.025	0.083	0.045	0.260	0.314	0.003
	K3	0.178	0.131	0.023	0.926	0.054	0.011	0.101	0.083	0.833	0.173	0.012	0.533
	Ca3	0.221	0.801	0.670	<0.0005	<0.0005	0.204	0.025	0.061	0.461	0.139	0.224	0.151
	Ti3	0.221	0.705	0.922	<0.0005	0.581	<0.0005	0.456	0.083	0.027	0.086	1.000	0.662
	V3	0.624	0.571	0.108	0.404	0.003	<0.0005	0.025	0.009	0.002	0.066	0.048	0.662
	Cr3	0.806	0.659	0.002	0.430	0.917	0.699	0.025	0.021	0.206	0.139	0.015	0.034
	Mn3	0.713	0.284	0.622	<0.0005	<0.0005	0.128	0.025	0.021	0.598	0.441	0.211	0.454
	Fe3	0.178	0.529	0.053	0.265	0.066	0.005	0.101	0.312	0.343	0.374	0.034	0.318
	Co3	0.111	0.753	0.071	<0.0005	<0.0005	<0.0005	0.025	0.083	0.002	0.441	0.193	0.662
	Ni3	0.270	0.571	0.670	<0.0005	<0.0005	0.002	0.025	0.773	0.020	0.086	0.089	0.851
	Cu3	0.806	0.850	0.309	<0.0005	<0.0005	0.117	0.025	0.386	0.171	0.515	0.199	0.708
	Zn3	0.624	0.753	0.178	<0.0005	<0.0005	0.427	0.025	0.083	0.792	0.110	0.126	0.851
	Sr3	0.391	0.529	0.108	<0.0005	0.008	0.180	0.025	0.021	0.598	0.173	0.211	0.289
	Mo3	0.540	0.529	0.470	<0.0005	0.001	0.100	0.025	0.194	0.114	0.859	0.251	0.755
	Ba3	0.270	0.450	0.082	<0.0005	<0.0005	0.785	0.881	0.564	0.343	0.260	<0.0005	0.105
	Cd3	0.391	0.875	0.026	<0.0005	<0.0005	0.955	0.025	0.009	0.916	0.767	0.754	0.512
	Pb3	0.540	0.950	0.818	<0.0005	<0.0005	0.928	0.180	0.386	0.527	0.110	0.102	0.574

F4	Na4	0.624	0.006	0.001	0.001	<0.0005	0.002	0.456	0.014	0.002	0.015	0.001	0.001
	Mg4	0.111	0.801	0.718	0.048	0.030	0.340	0.025	0.009	0.461	0.015	0.754	0.755
	Al4	0.540	0.950	0.870	0.853	0.917	0.540	0.297	0.083	0.673	0.173	0.266	0.492
	Si4	0.540	0.659	0.533	<0.0005	<0.0005	0.716	0.025	0.009	0.673	0.028	0.917	0.803
	K4	1.000	0.705	0.718	0.011	0.013	0.340	0.456	0.470	0.916	0.515	0.651	0.289
	Ca4	0.462	0.900	0.577	<0.0005	0.022	0.143	0.881	0.773	0.833	0.594	0.808	0.086
	Ti4	0.903	0.614	0.718	<0.0005	0.011	0.785	0.025	0.248	0.461	0.859	0.835	0.382
	Mn4	1.000	0.488	0.450	<0.0005	<0.0005	0.220	0.025	0.051	0.246	0.594	0.237	0.755
	Fe4	0.806	0.614	0.670	0.404	0.139	0.525	0.025	0.014	0.206	0.021	0.237	0.212
	P4	0.327	0.186	0.028	<0.0005	<0.0005	<0.0005	0.025	0.564	0.002	0.015	0.348	0.261
	S4	0.540	0.166	0.200	<0.0005	<0.0005	0.123	0.180	0.194	0.399	0.173	<0.0005	0.003
	Cr4	1.000	0.753	0.309	<0.0005	0.008	0.683	0.025	0.061	0.833	0.173	0.677	0.662
	V4	0.806	0.900	0.922	0.009	0.215	0.803	0.456	0.470	0.916	0.214	0.945	0.492
	Co4	0.178	0.571	0.577	<0.0005	0.014	0.014	0.025	0.009	0.092	0.314	0.728	0.950
	Ni4	0.270	0.115	0.039	<0.0005	<0.0005	0.683	0.025	0.009	0.461	0.086	0.808	0.261
	Cu4	0.391	0.014	0.622	<0.0005	<0.0005	0.388	0.025	0.009	0.527	0.110	0.781	0.289
	Zn4	0.221	0.801	0.450	0.010	0.429	0.467	0.456	0.083	0.082	0.260	0.118	0.119
	Sr4	0.221	0.900	0.622	<0.0005	<0.0005	0.964	0.025	0.009	0.916	0.028	0.728	0.170
	Ba4	0.086	0.801	0.412	<0.0005	<0.0005	0.256	0.025	0.009	0.045	0.011	0.331	0.046
	Pb4	0.327	0.257	0.974	0.002	0.038	0.204	0.456	0.885	0.527	0.374	0.555	0.492

Mobility, %	Na_m	0.111	0.018	0.030	0.781	0.754	0.964	0.025	0.061	1.000	0.554	<0.0005	0.001
	Mg_m	0.540	0.571	0.577	<0.0005	<0.0005	0.102	0.025	0.009	0.171	0.214	0.945	0.950
	Al_m	1.000	0.147	0.053	<0.0005	0.777	<0.0005	0.456	0.030	0.002	0.021	0.424	0.492
	Si_m	0.540	0.413	0.622	0.009	0.001	0.069	0.053	0.009	0.011	0.086	0.071	0.901
	K_m	0.903	0.450	0.001	<0.0005	<0.0005	0.716	0.456	0.386	0.833	0.038	0.154	0.289
	Ca_m	0.327	0.850	0.577	<0.0005	<0.0005	0.184	0.025	0.112	0.673	0.066	0.651	0.086
	Ti_m	0.391	0.488	0.974	<0.0005	0.110	0.021	0.297	0.885	0.292	0.051	0.702	0.618
	Mn_m	1.000	0.614	0.412	<0.0005	<0.0005	0.570	0.025	0.051	0.598	0.594	0.281	0.851
	Fe_m	0.221	0.284	0.033	0.228	<0.0005	<0.0005	0.053	0.009	0.020	0.021	0.135	0.289
	P_m	0.270	0.284	0.017	<0.0005	<0.0005	<0.0005	0.025	0.149	0.002	0.767	0.781	0.349
	S_m	0.713	0.378	0.011	<0.0005	<0.0005	0.004	0.025	0.194	0.058	0.173	0.945	0.004
	Cr_m	0.462	0.186	0.061	0.010	0.004	0.555	0.025	0.009	0.206	0.038	0.001	0.289
	V_m	0.270	0.950	0.094	0.073	0.001	<0.0005	0.025	0.009	0.002	0.021	0.018	0.618
	Co_m	0.111	0.314	0.577	<0.0005	<0.0005	<0.0005	0.025	0.014	0.073	0.441	0.651	0.533
	Ni_m	0.111	0.488	0.033	<0.0005	<0.0005	0.173	0.025	0.014	0.206	0.515	0.331	0.901
	Cu_m	0.327	0.529	0.309	<0.0005	<0.0005	0.033	0.025	0.009	0.246	0.314	0.366	0.901
	Zn_m	0.540	0.850	0.014	<0.0005	<0.0005	0.089	0.025	0.009	0.461	0.953	0.102	0.289
	Sr_m	0.221	0.284	0.200	<0.0005	<0.0005	0.733	0.025	0.009	0.673	0.038	0.808	0.212
	Ba_m	0.713	0.208	0.491	<0.0005	<0.0005	0.910	0.456	0.021	0.002	0.038	0.065	0.002
	Pb_m	0.391	0.166	0.818	0.877	0.174	0.153	0.655	0.885	0.752	0.515	0.862	0.382

T – topsoil, S – subsoil, PM – parent material.

The ChE mobility (ChE_m, Table S1) was calculated as a ratio of its mobile fractions (F1+F2+F3) to its total content, multiplied by 100%.

At the MSU Faculty of Geology, soil mineralogical composition (phyllosilicates and other minerals; Table 4) was determined using an ULTIMA IV X-Ray diffractometer (made by Rigaku, Japan) operated at 40 kV, 40 mA, 3–65° 2θ, with Cu radiation and a DTex/Ultra semiconductor detector. Minerals were identified by comparing experimental data with standard X-Ray patterns from the PDF-2 database with the use of the MDI Jade 6.5 software and methodological recommendations by [12], [13], [14]. A quantitative mineralogical analysis was carried out using the Rietveld full-pattern fitting method [15] and the BGMN software [16].

Statistical analyses included calculations of descriptive statistics (Table S1), a Spearman rank ratio (Table 5). For different datasets, a Mann-Whitney U-test was conducted (Table 6).

## CRediT authorship contribution statement

**Ivan N. Semenkov:** Writing – original draft, Writing – review & editing, Data curation, Investigation, Conceptualization, Methodology, Visualization. **Maria V. Konyushkova:** Supervision, Writing – review & editing. **Galya V. Klink:** Visualization, Investigation. **Victoria V. Krupskaya:** Methodology, Writing – review & editing. **Polina R. Enchilik:** Visualization, Writing – review & editing. **Nina M. Novikova:** Writing – review & editing, Visualization, Investigation.

## Declaration of Competing Interest

The authors declare that they have no known competing financial interests or personal relationships which have or could be perceived to have influenced the work reported in this article.

## Appendix Supplementary materials

Image, image 1

Image, image 2

Image, image 3

Image, image 4

Image, image 5

Image, image 6

Image, image 7

Image, image 8

Image, image 9

Image, image 10

Image, image 11

Image, image 12

Image, image 13

Image, image 14

Image, image 15

Image, image 16

Image, image 17

Image, image 18

Image, image 19

Image, image 20

Image, image 21

Image, image 22

Image, image 23

Image, image 24

Image, image 25

Image, image 26

Image, application 27
