# Supplementary figures and images for: Data on the soil and vegetation properties at the small gully catchment area: Steppe region of Kalmykia Republic (South Russia)
Source: Data Brief. 2021 Dec 24;40:107746. doi: 10.1016/j.dib.2021.107746 (PMC8717260; doi:10.1016/j.dib.2021.107746)

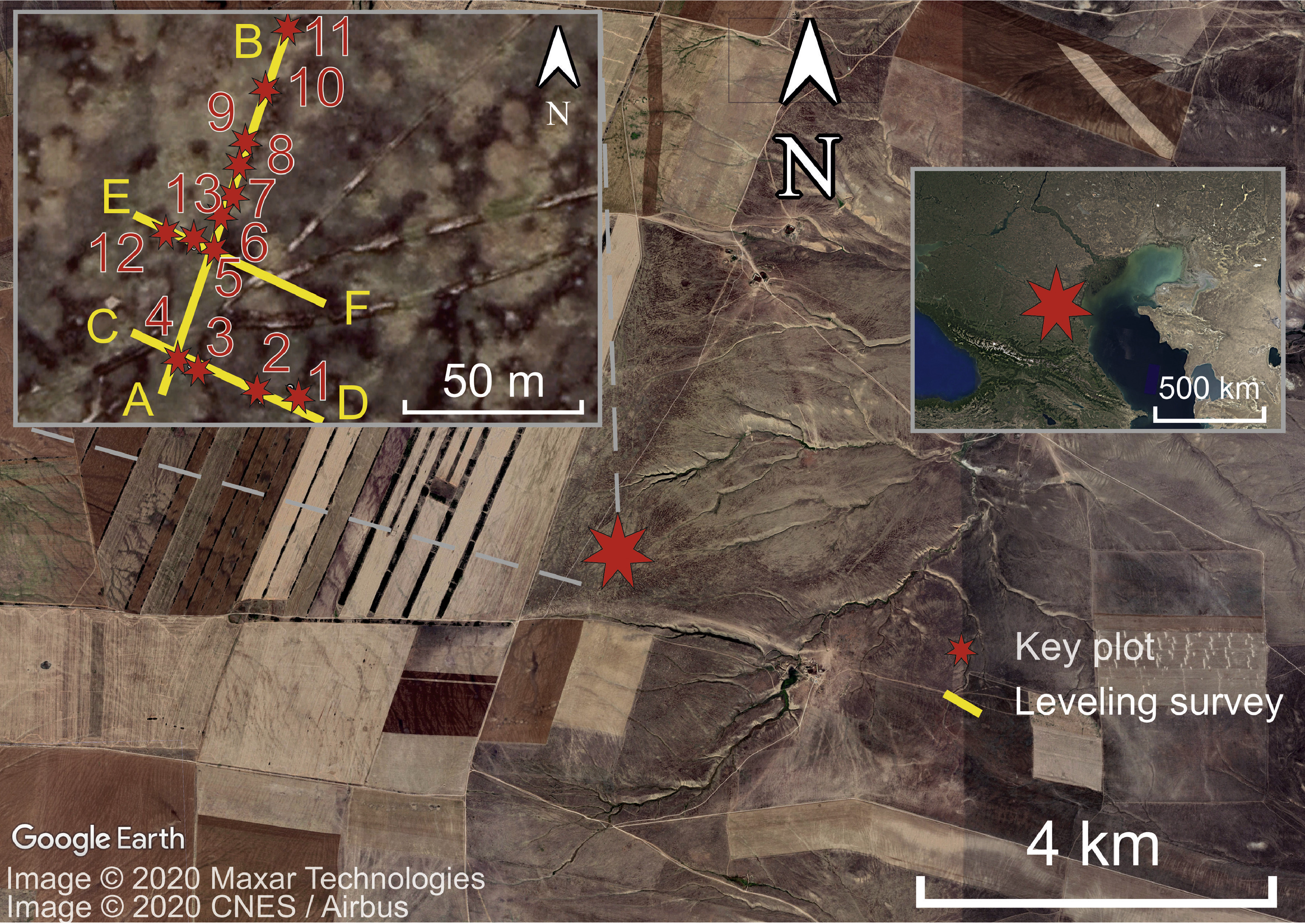

Supplement: Supplementary file 1 [file mmc1.jpg]

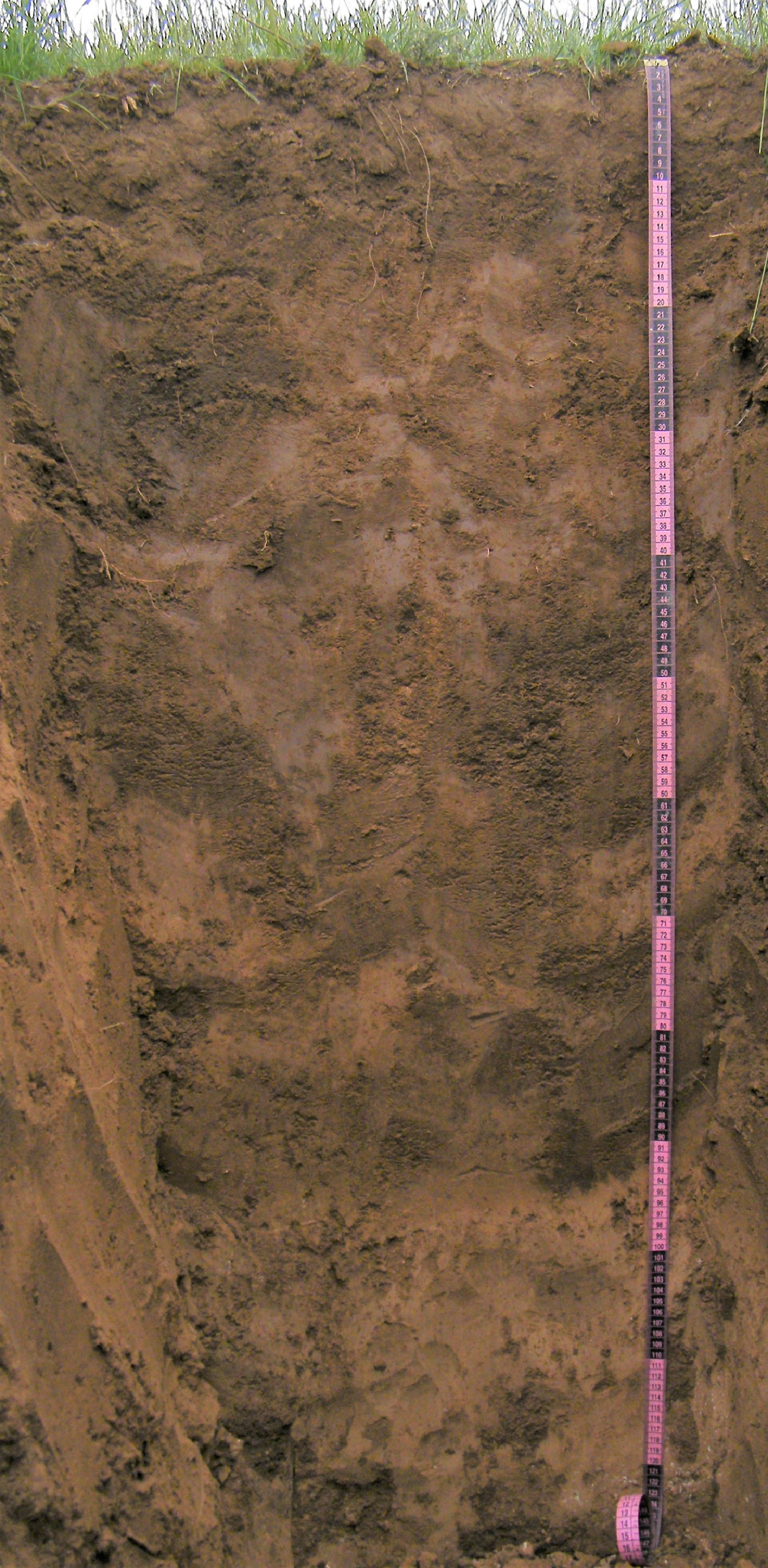

Supplement: Supplementary file 2 [file mmc2.jpg]

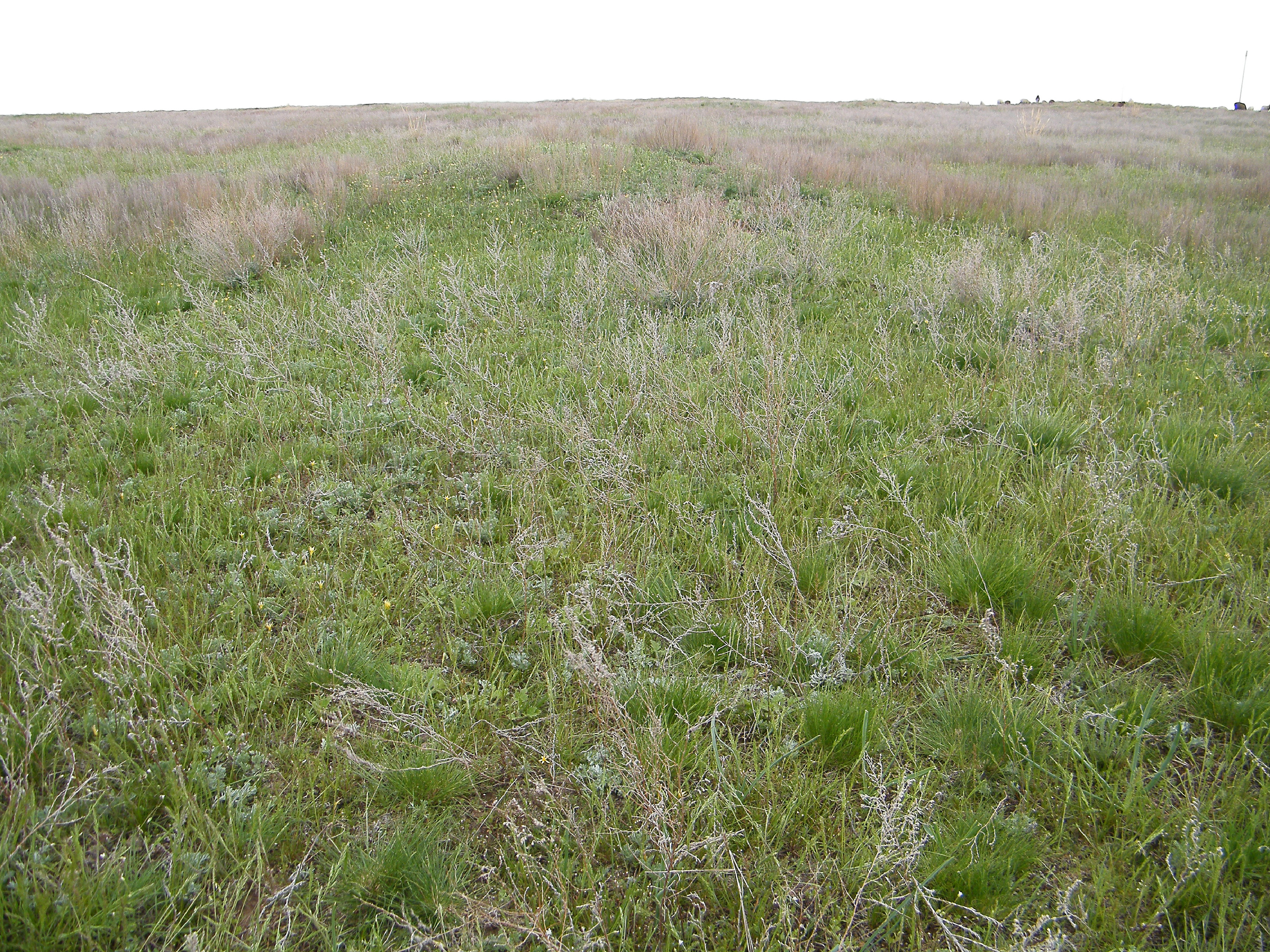

Supplement: Supplementary file 3 [file mmc3.jpg]

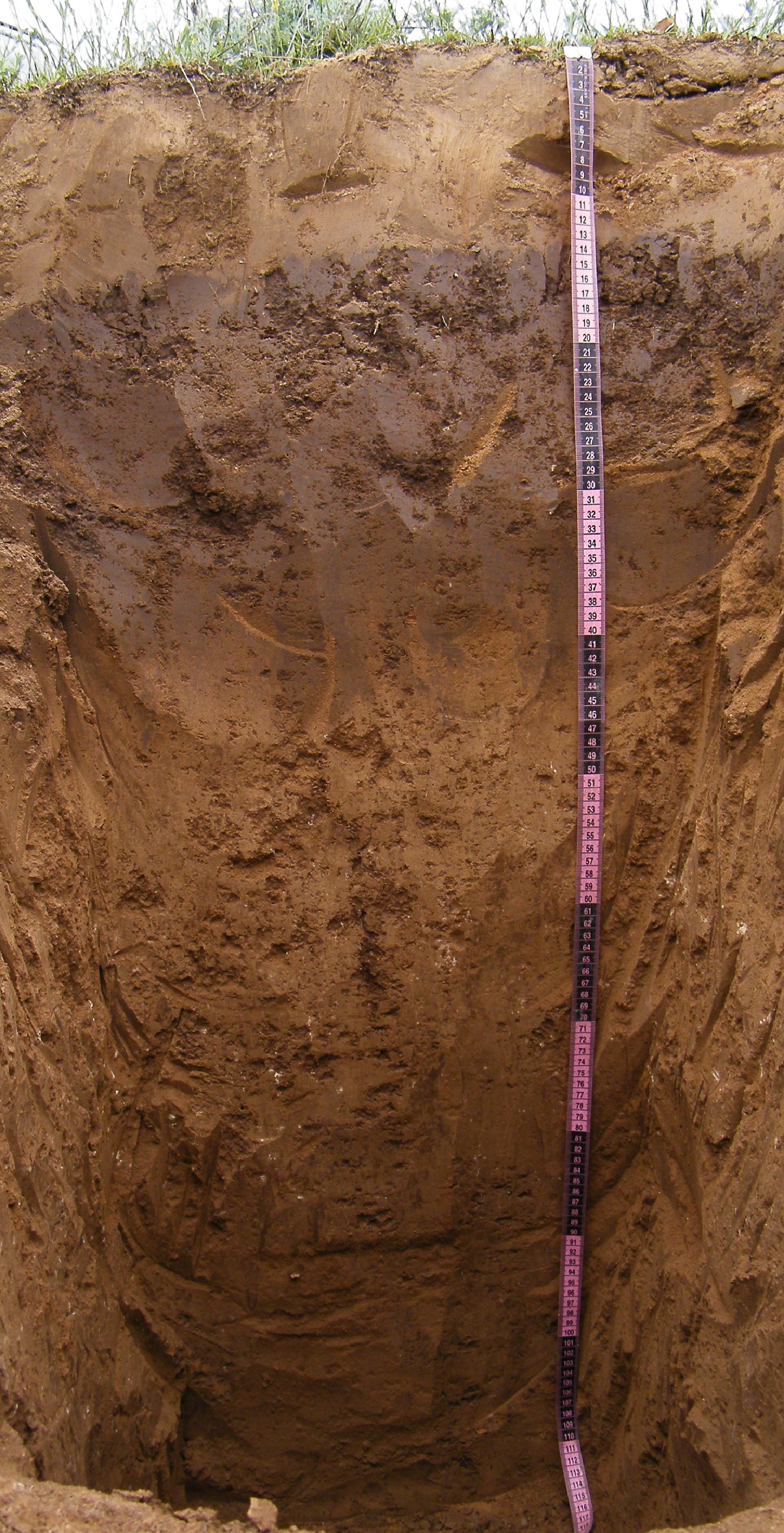

Supplement: Supplementary file 4 [file mmc4.jpg]

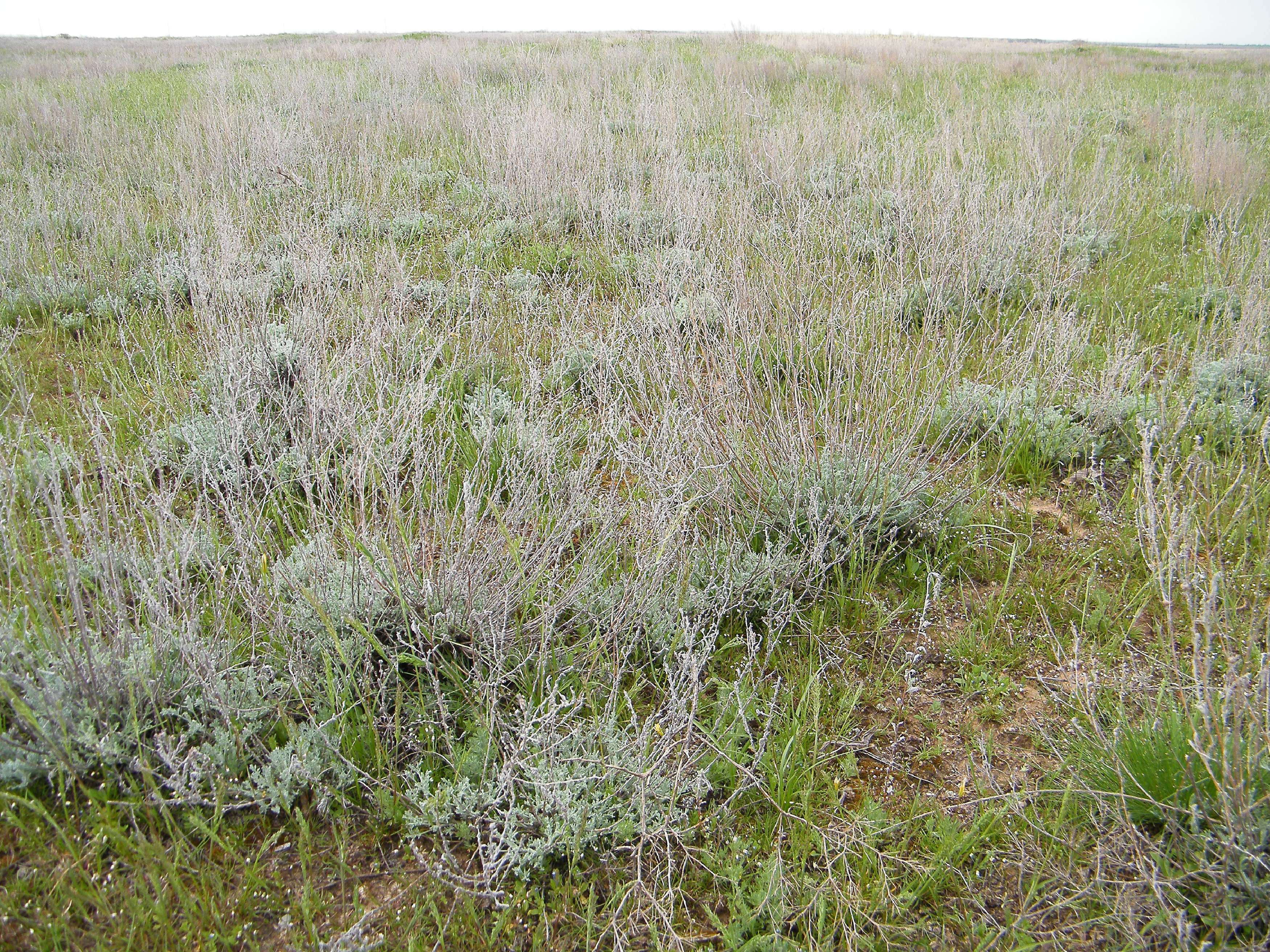

Supplement: Supplementary file 5 [file mmc5.jpg]

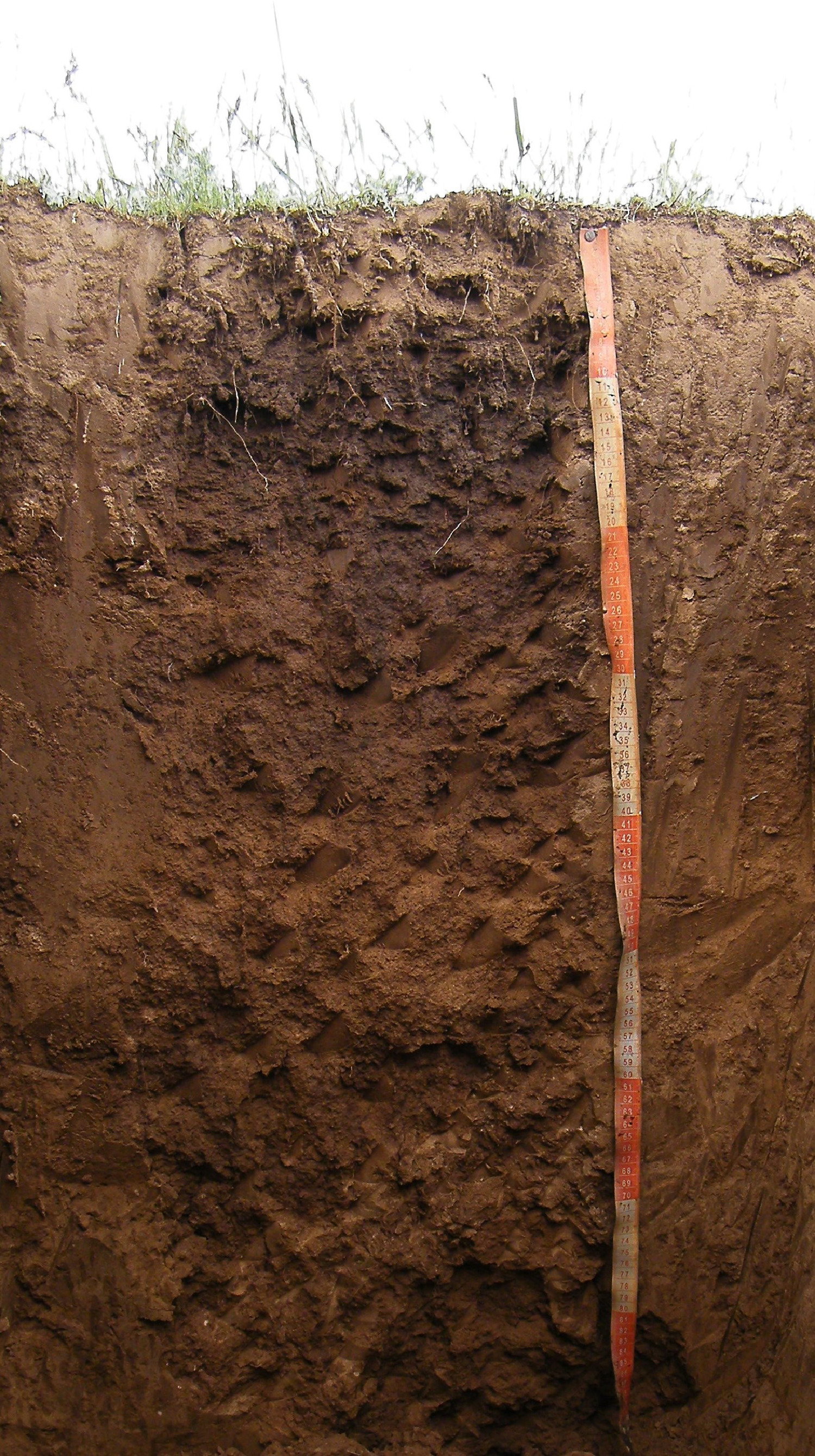

Supplement: Supplementary file 6 [file mmc6.jpg]

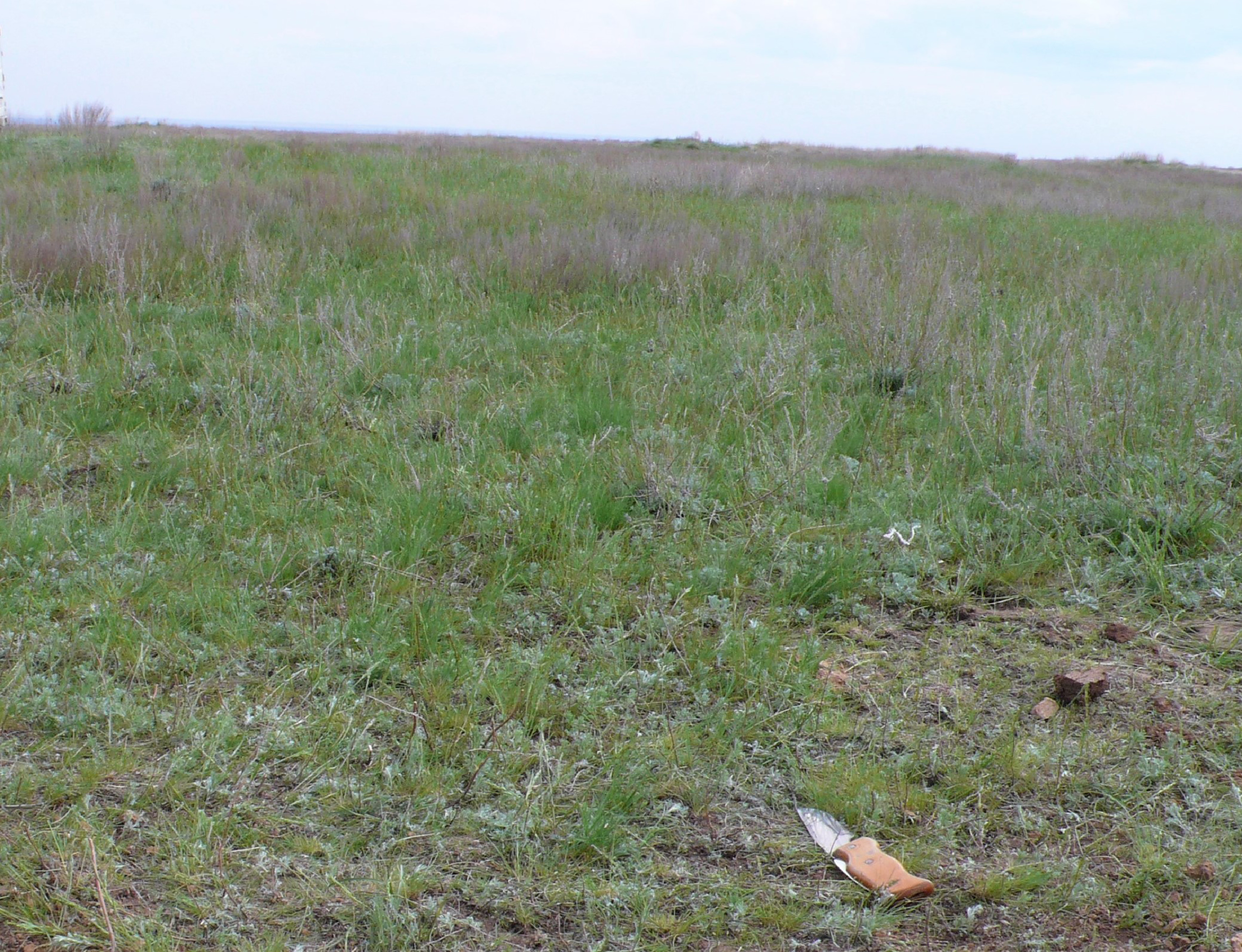

Supplement: Supplementary file 7 [file mmc7.jpg]

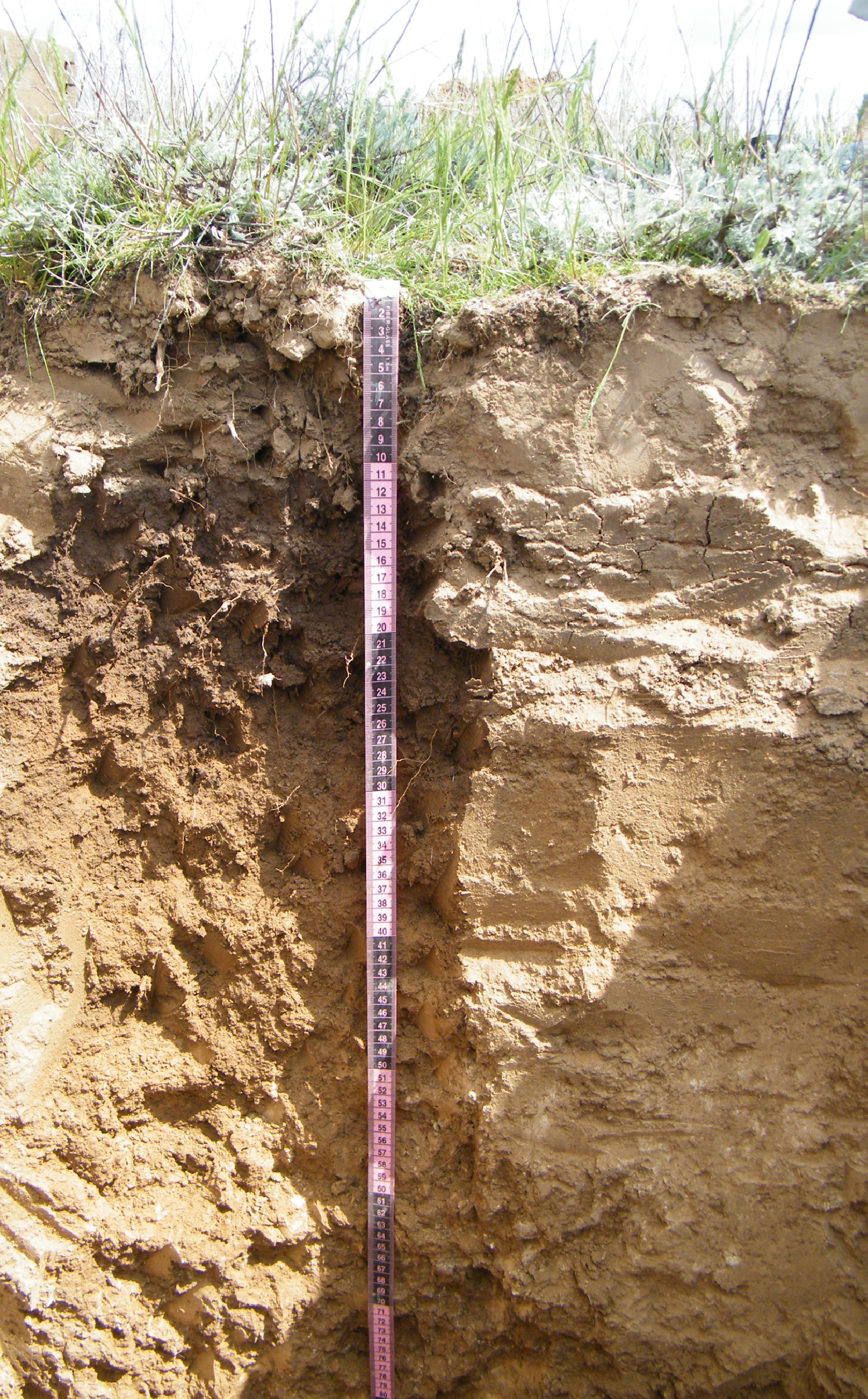

Supplement: Supplementary file 8 [file mmc8.jpg]

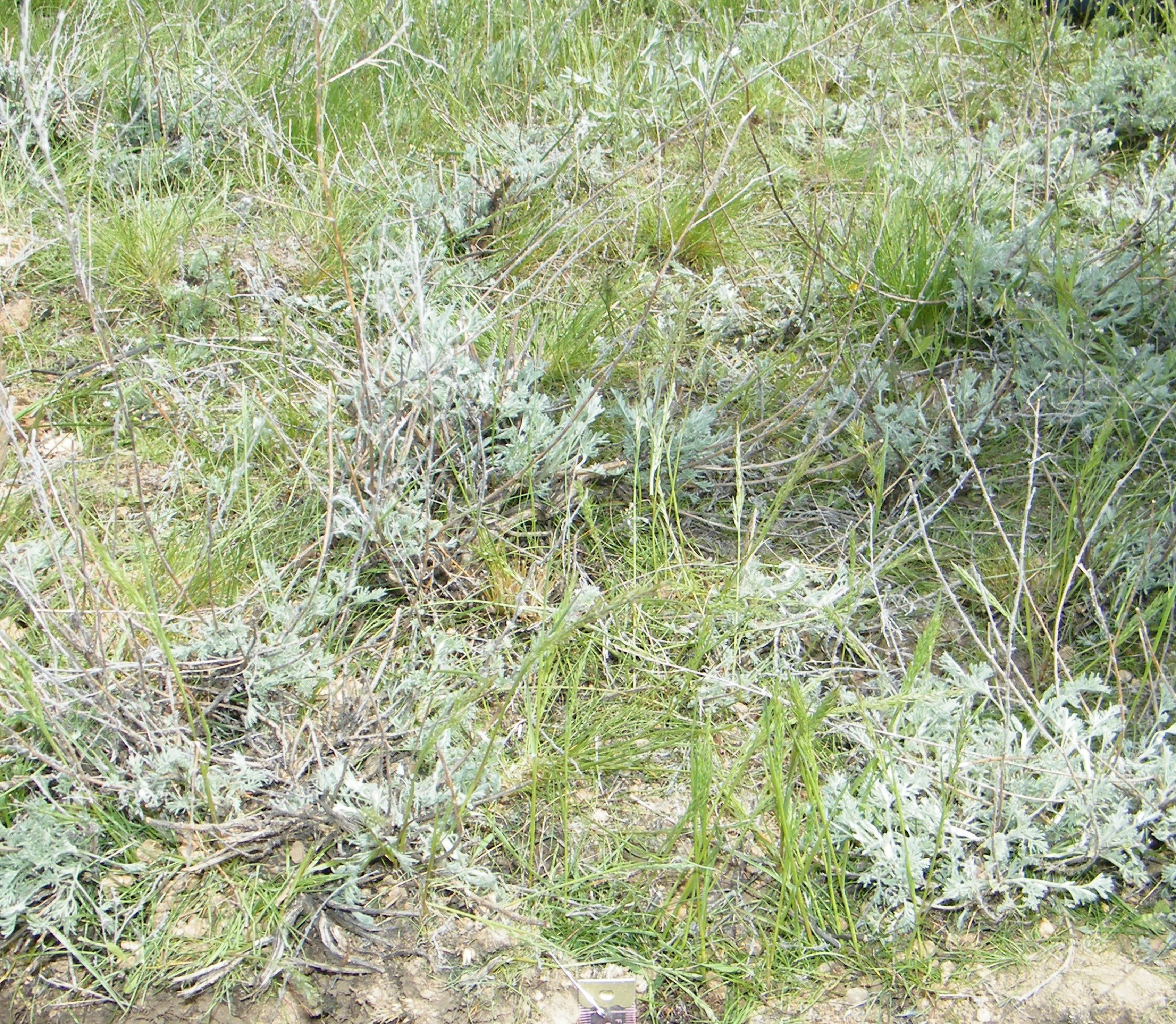

Supplement: Supplementary file 9 [file mmc9.jpg]

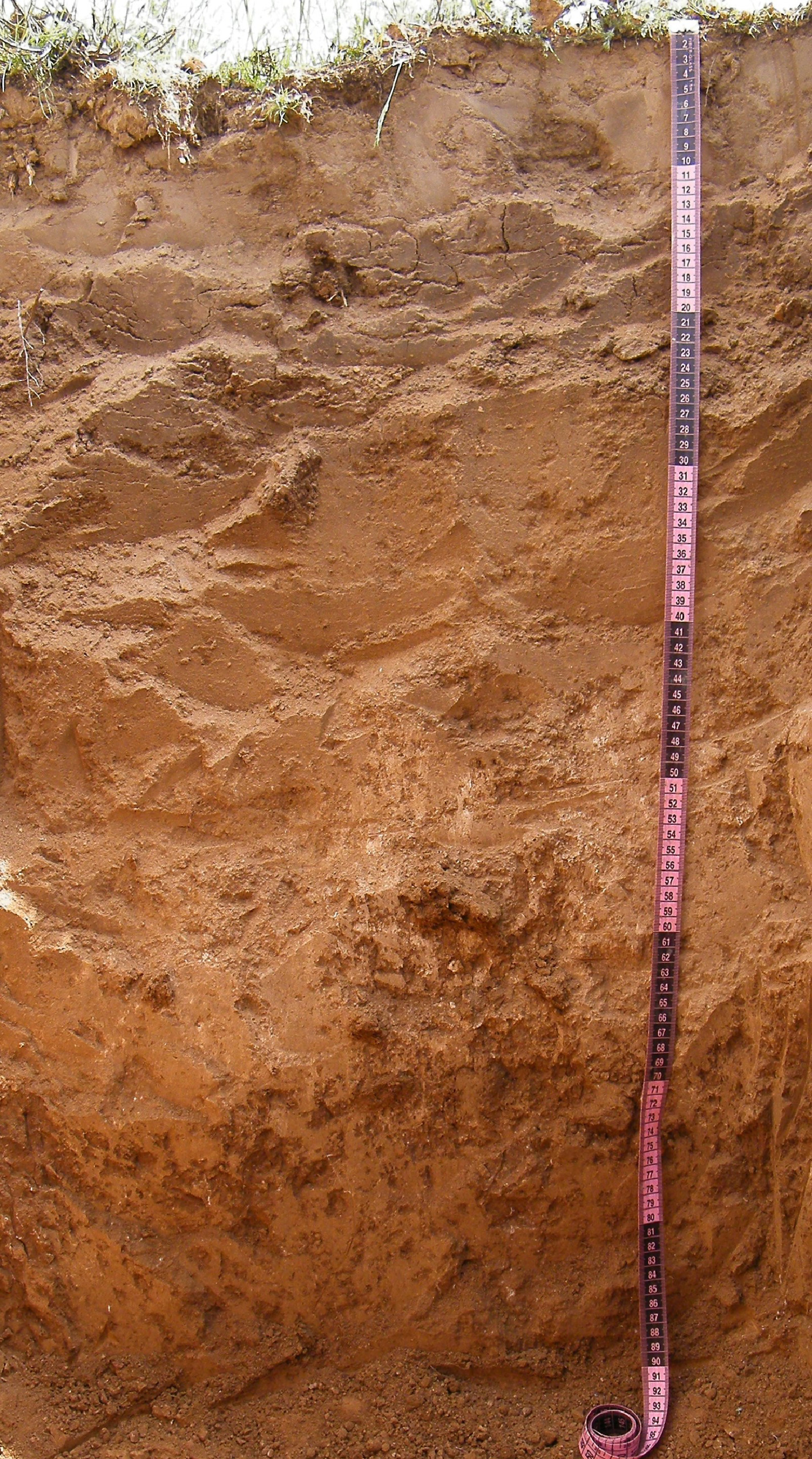

Supplement: Supplementary file 10 [file mmc10.jpg]

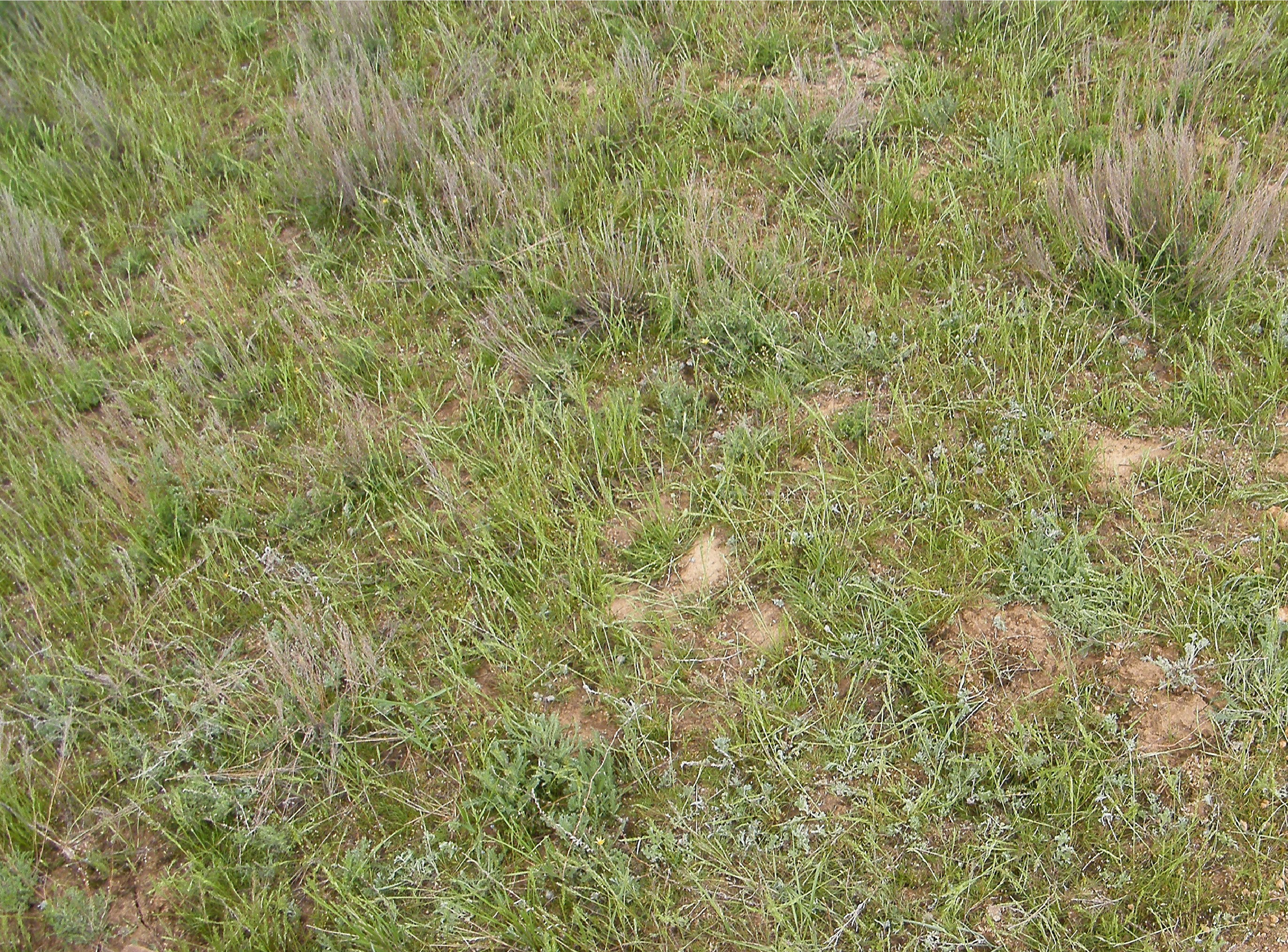

Supplement: Supplementary file 11 [file mmc11.jpg]

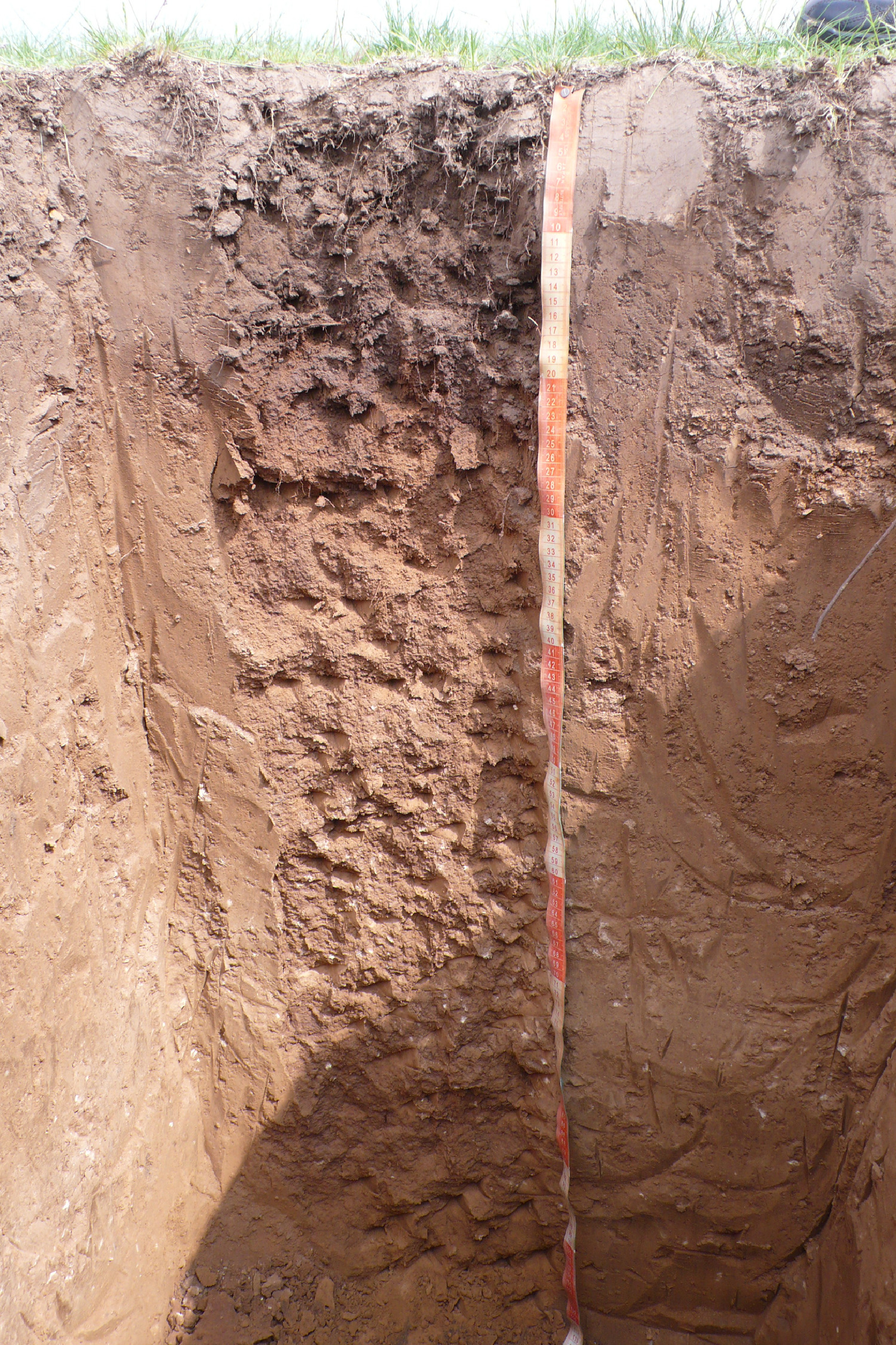

Supplement: Supplementary file 12 [file mmc12.jpg]

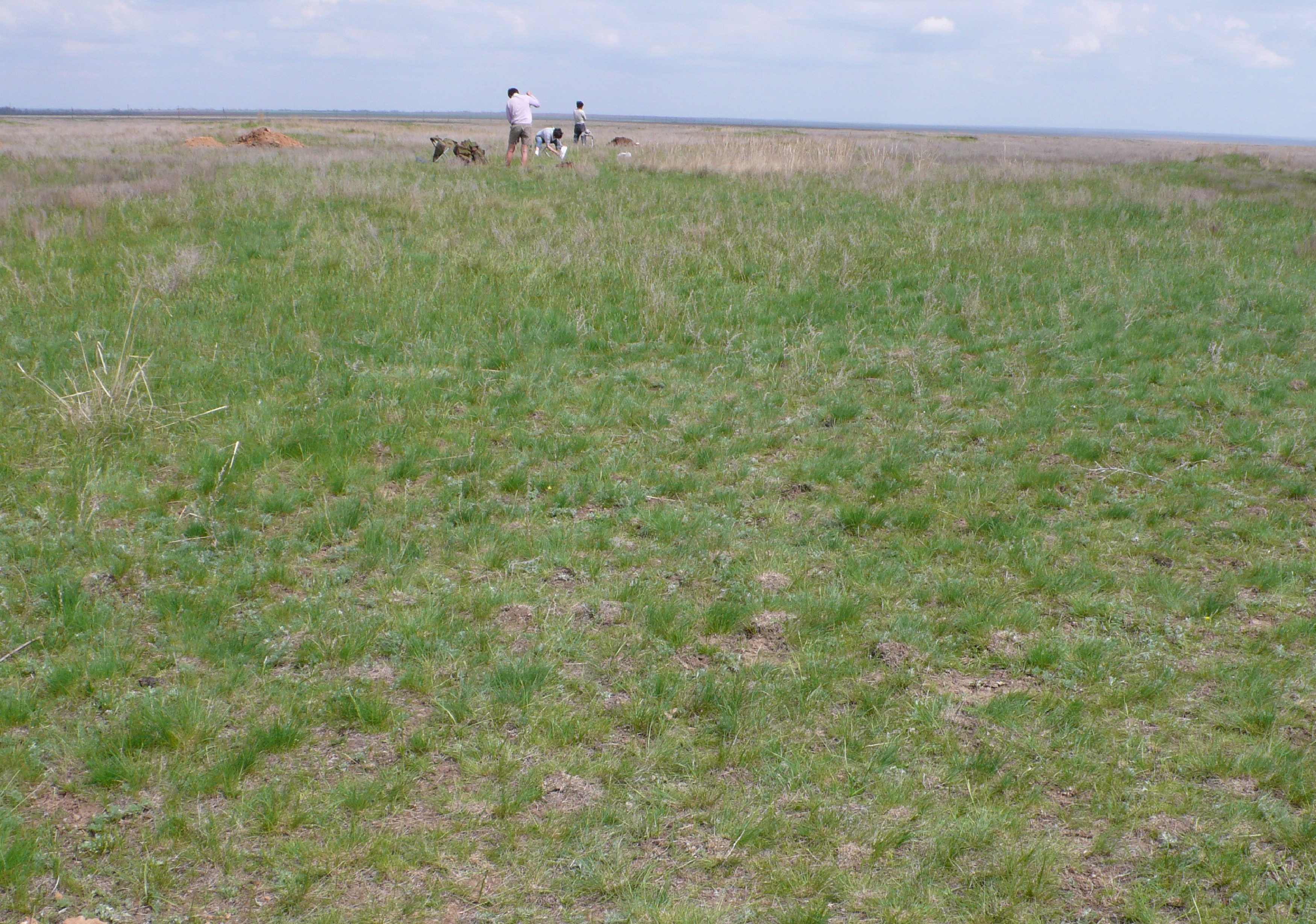

Supplement: Supplementary file 13 [file mmc13.jpg]

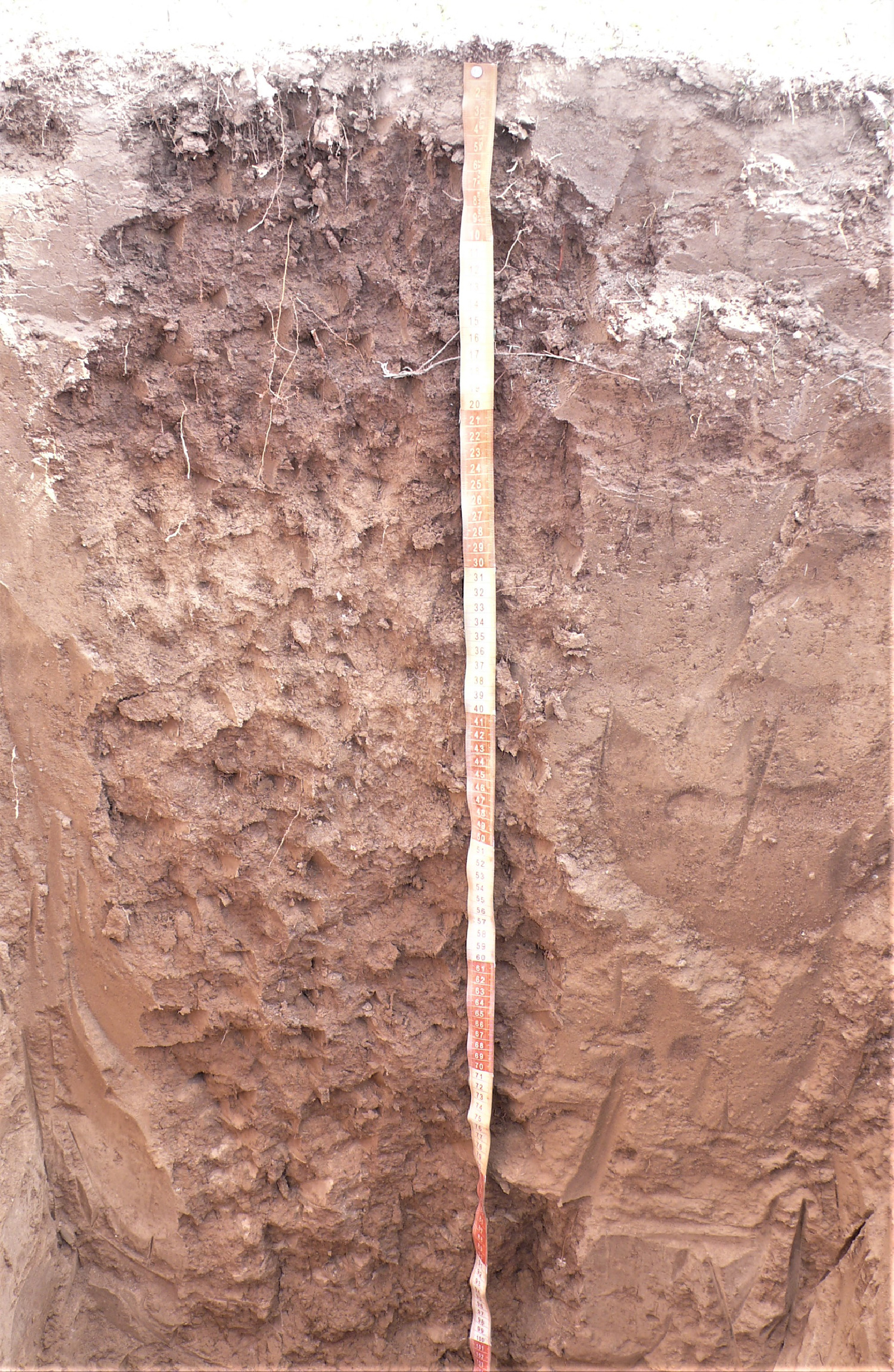

Supplement: Supplementary file 14 [file mmc14.jpg]

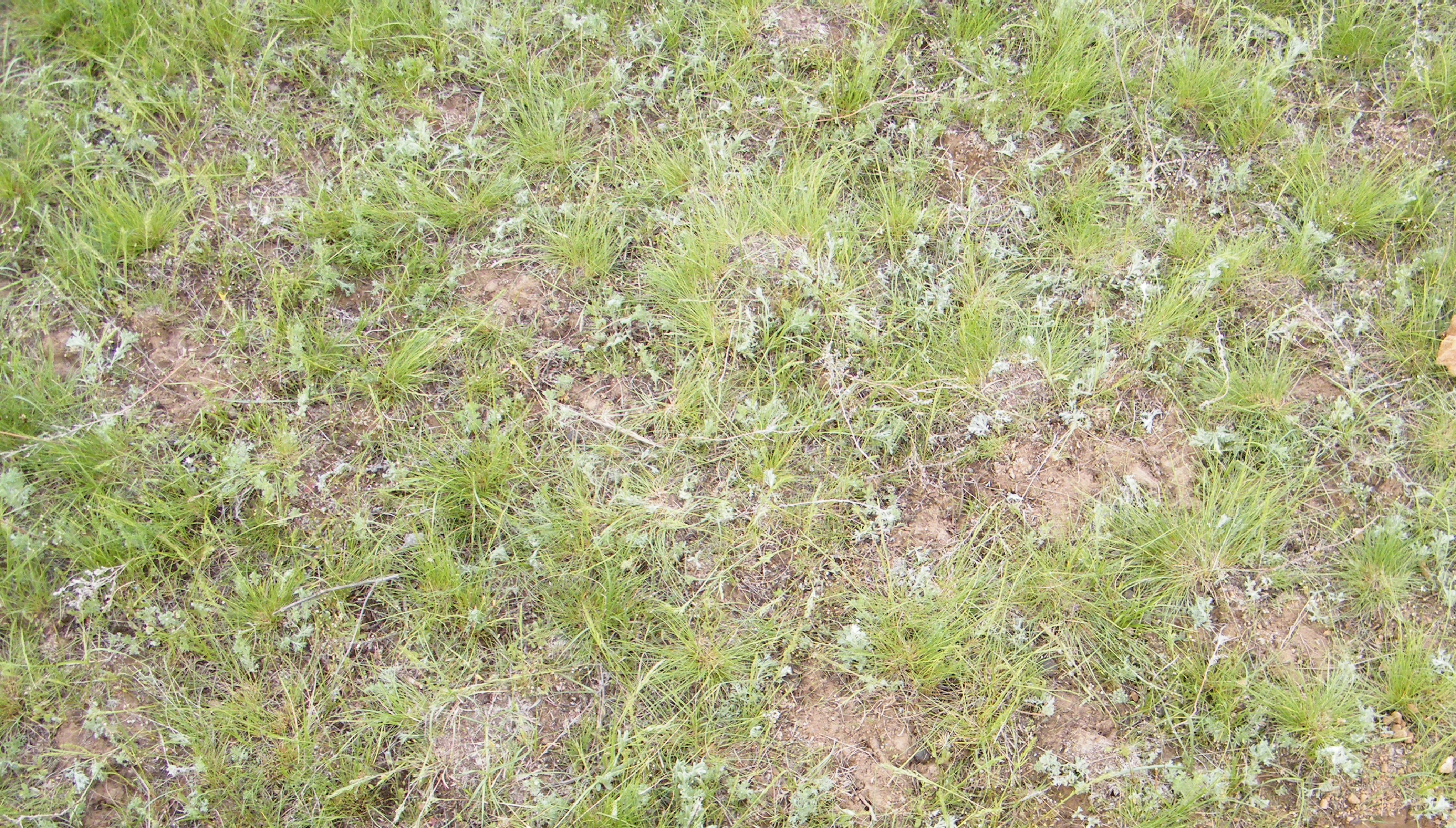

Supplement: Supplementary file 15 [file mmc15.jpg]

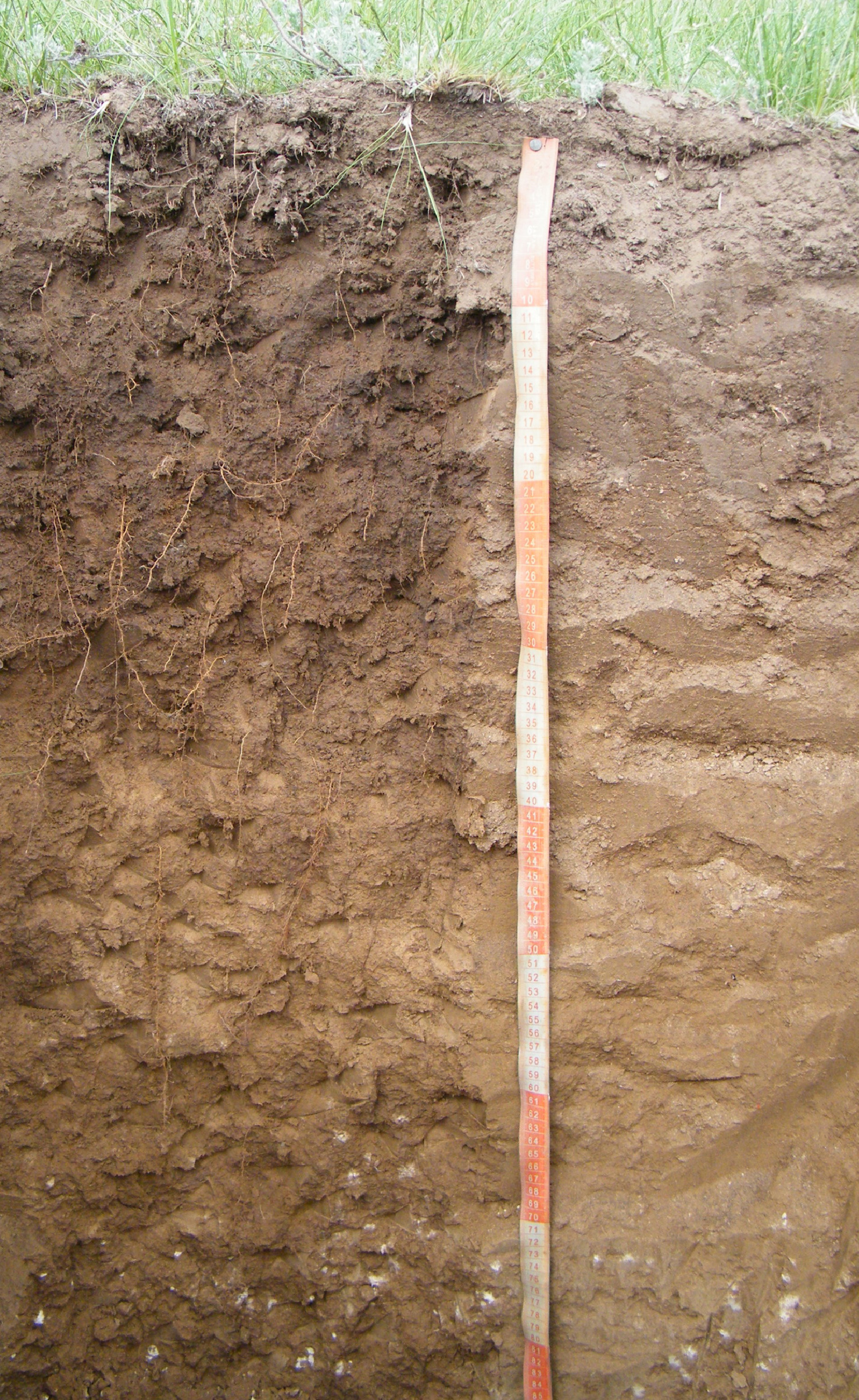

Supplement: Supplementary file 16 [file mmc16.jpg]

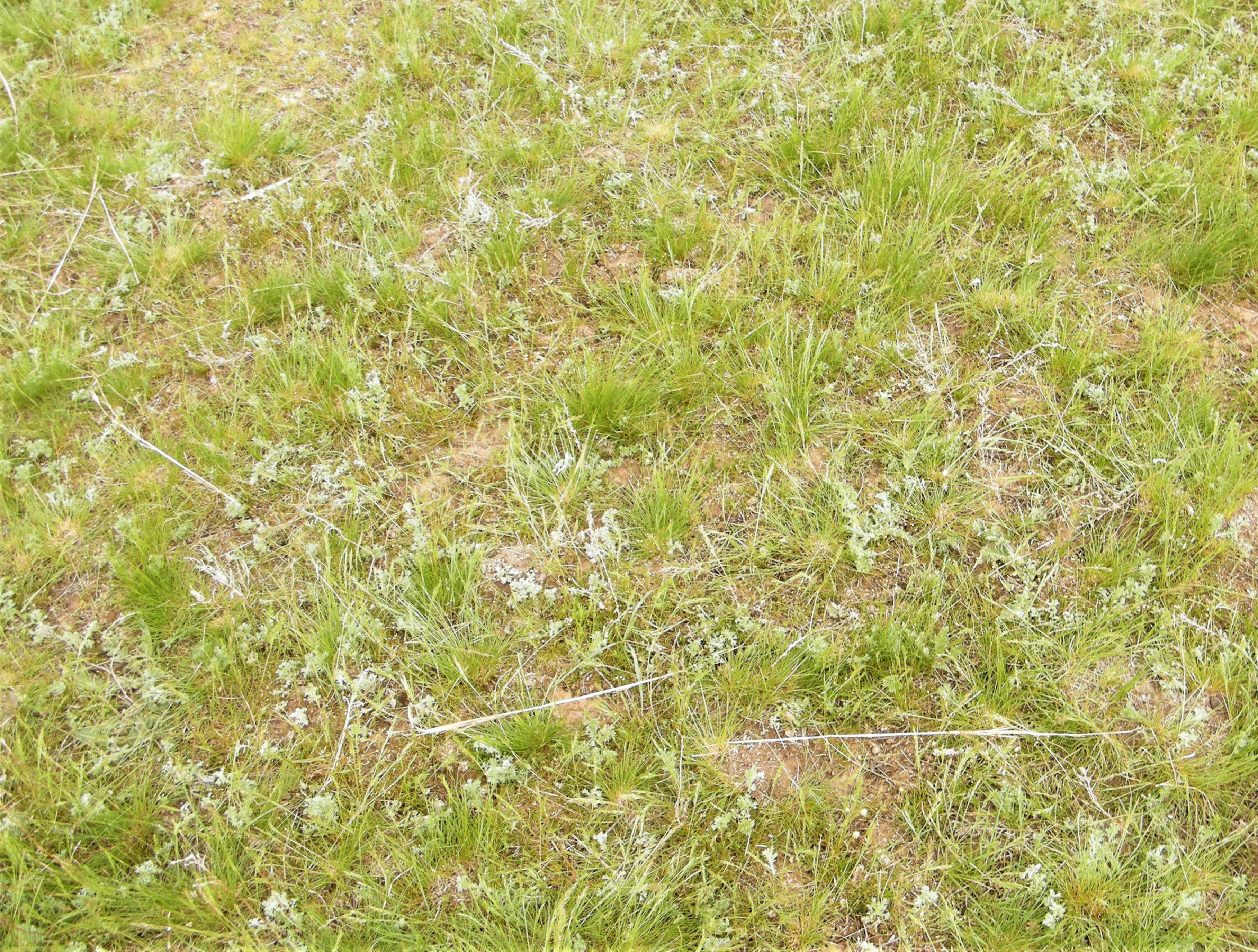

Supplement: Supplementary file 17 [file mmc17.jpg]

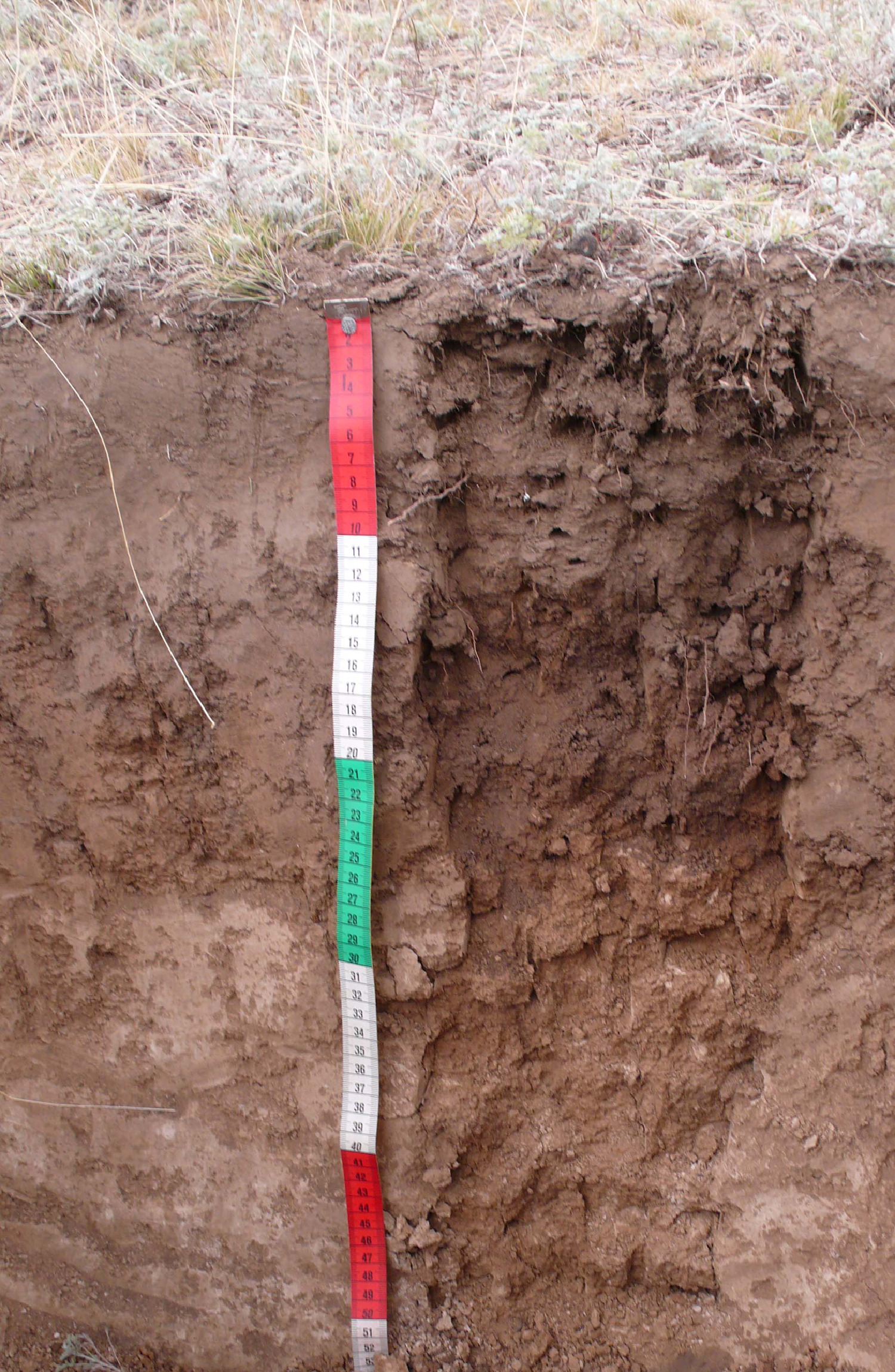

Supplement: Supplementary file 18 [file mmc18.jpg]

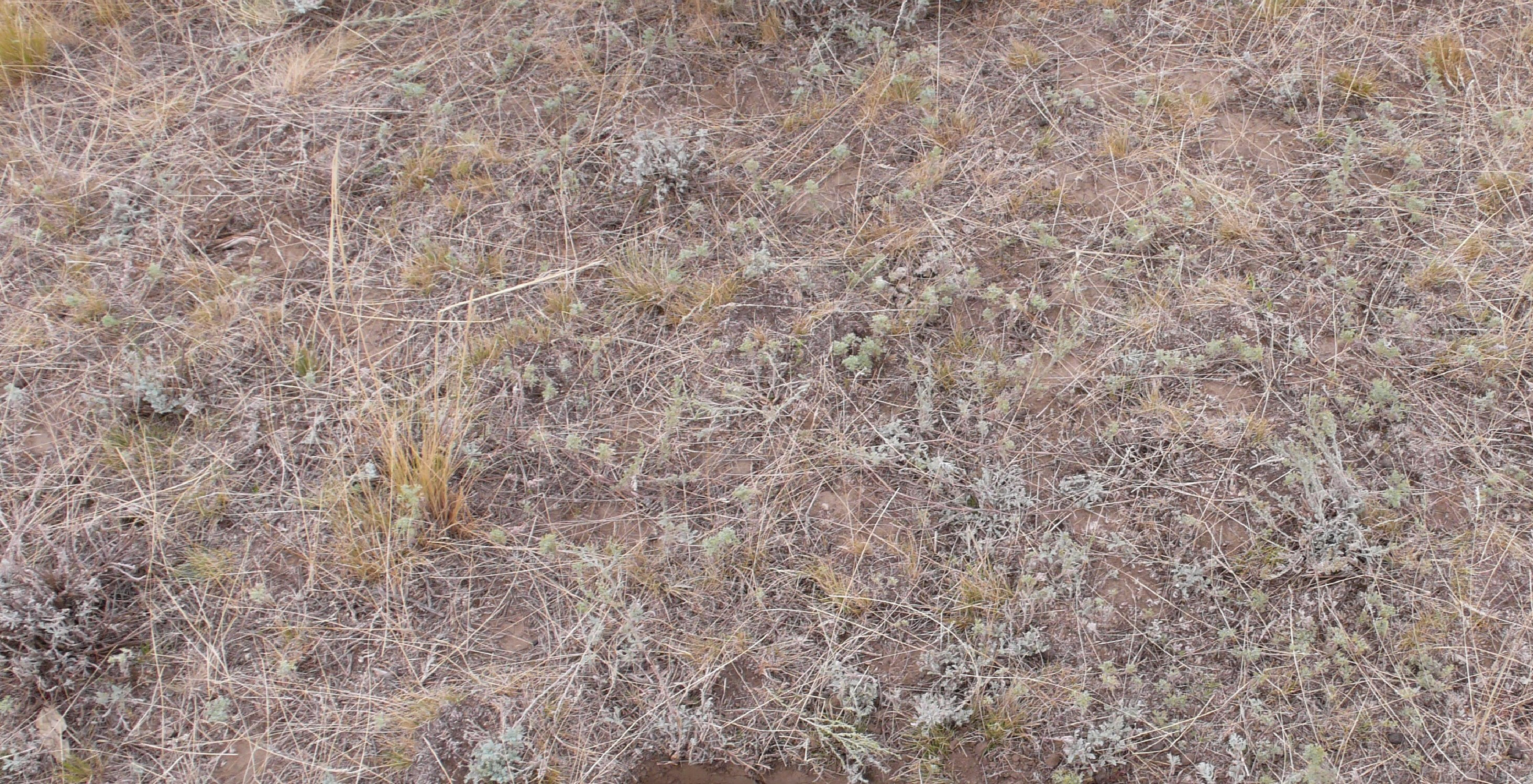

Supplement: Supplementary file 19 [file mmc19.jpg]

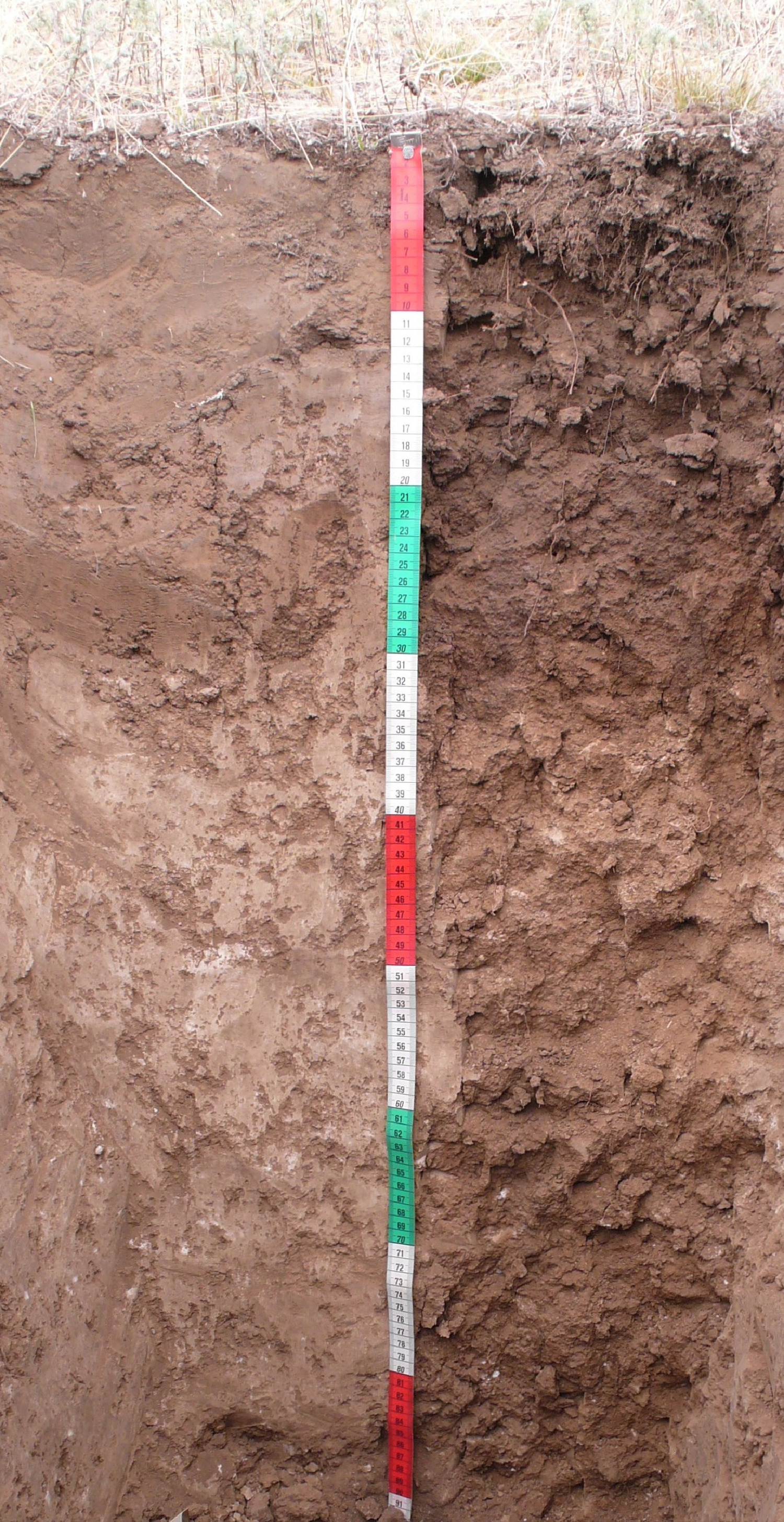

Supplement: Supplementary file 20 [file mmc20.jpg]

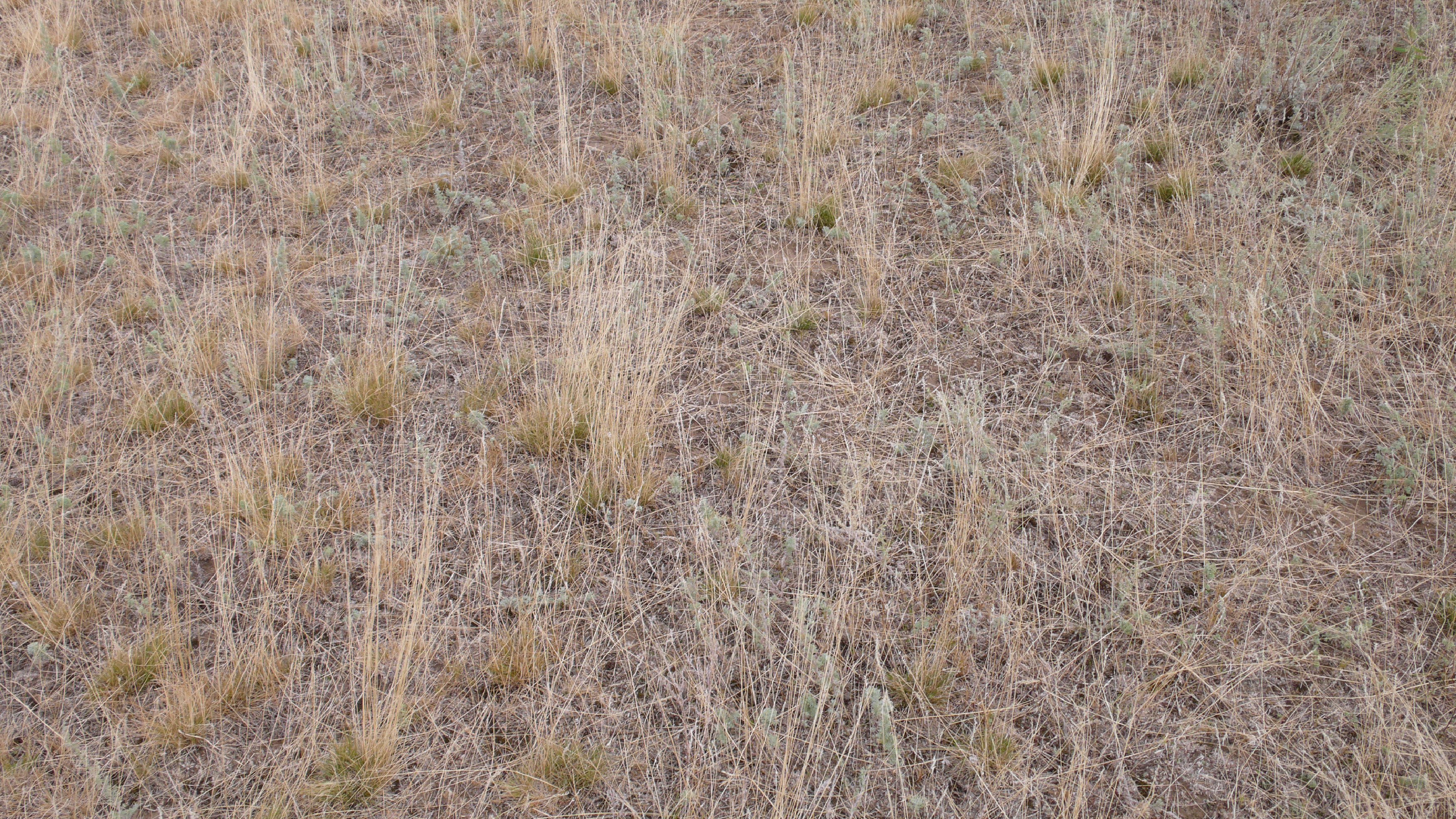

Supplement: Supplementary file 21 [file mmc21.jpg]

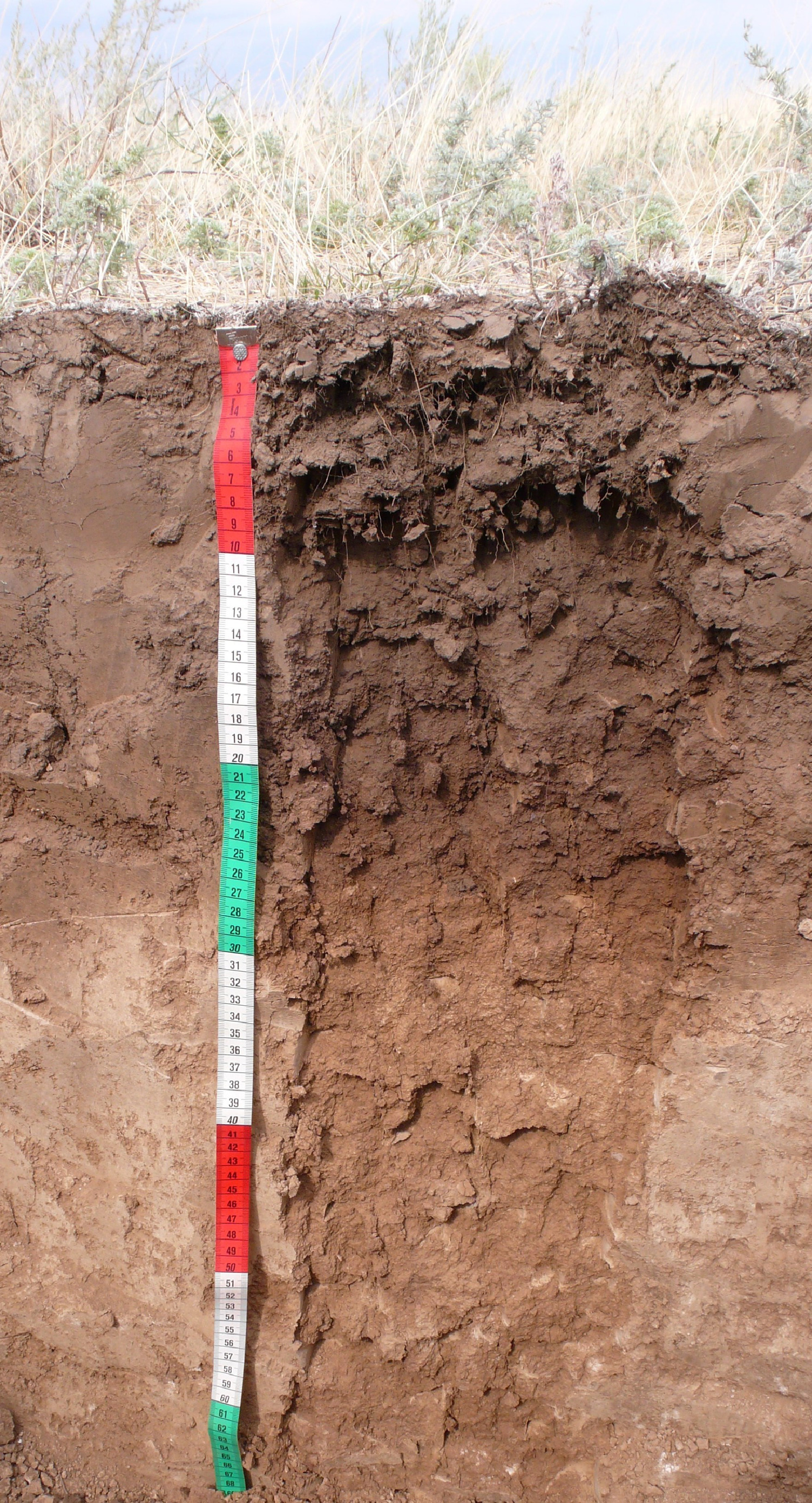

Supplement: Supplementary file 22 [file mmc22.jpg]

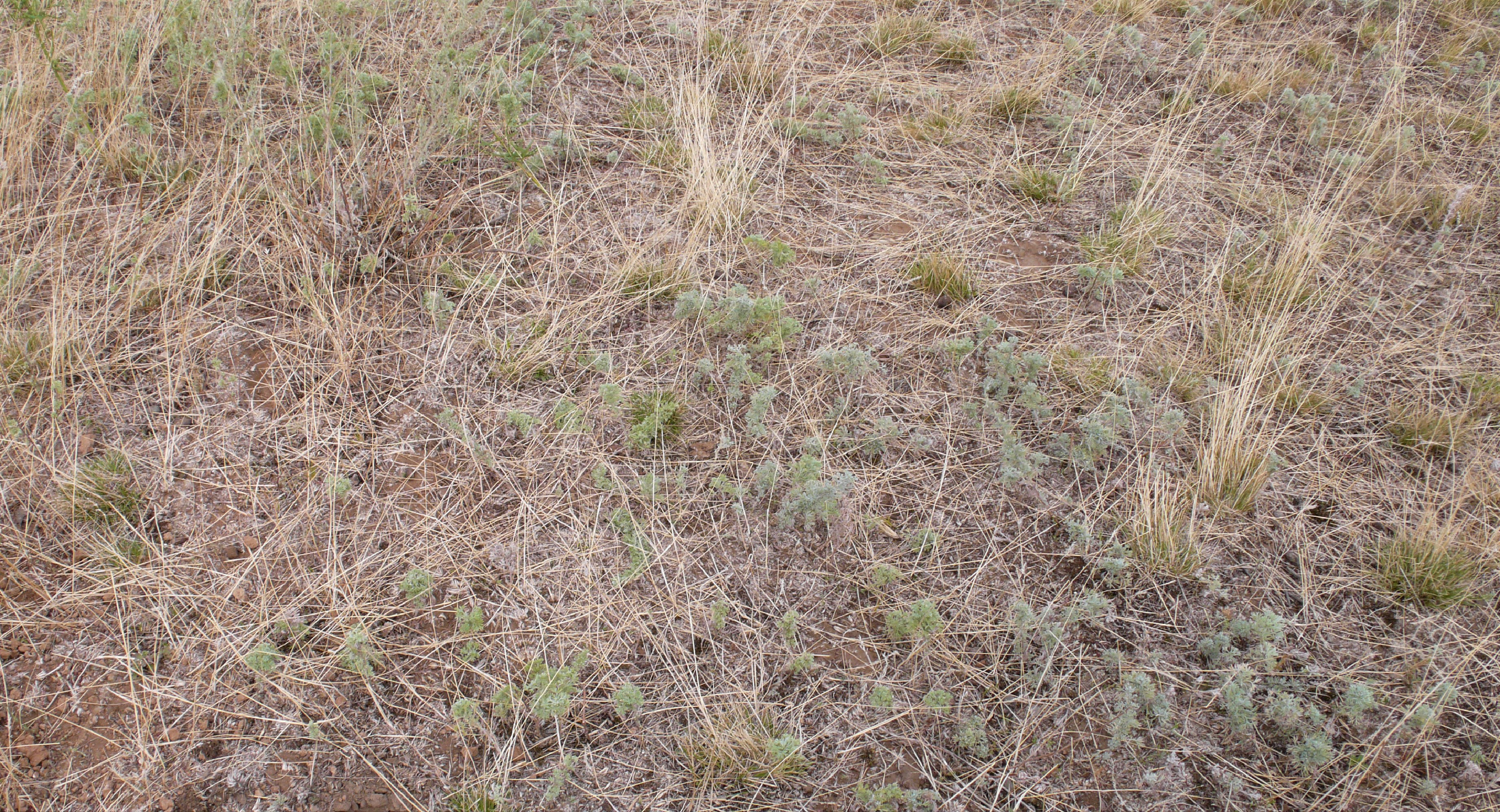

Supplement: Supplementary file 23 [file mmc23.jpg]

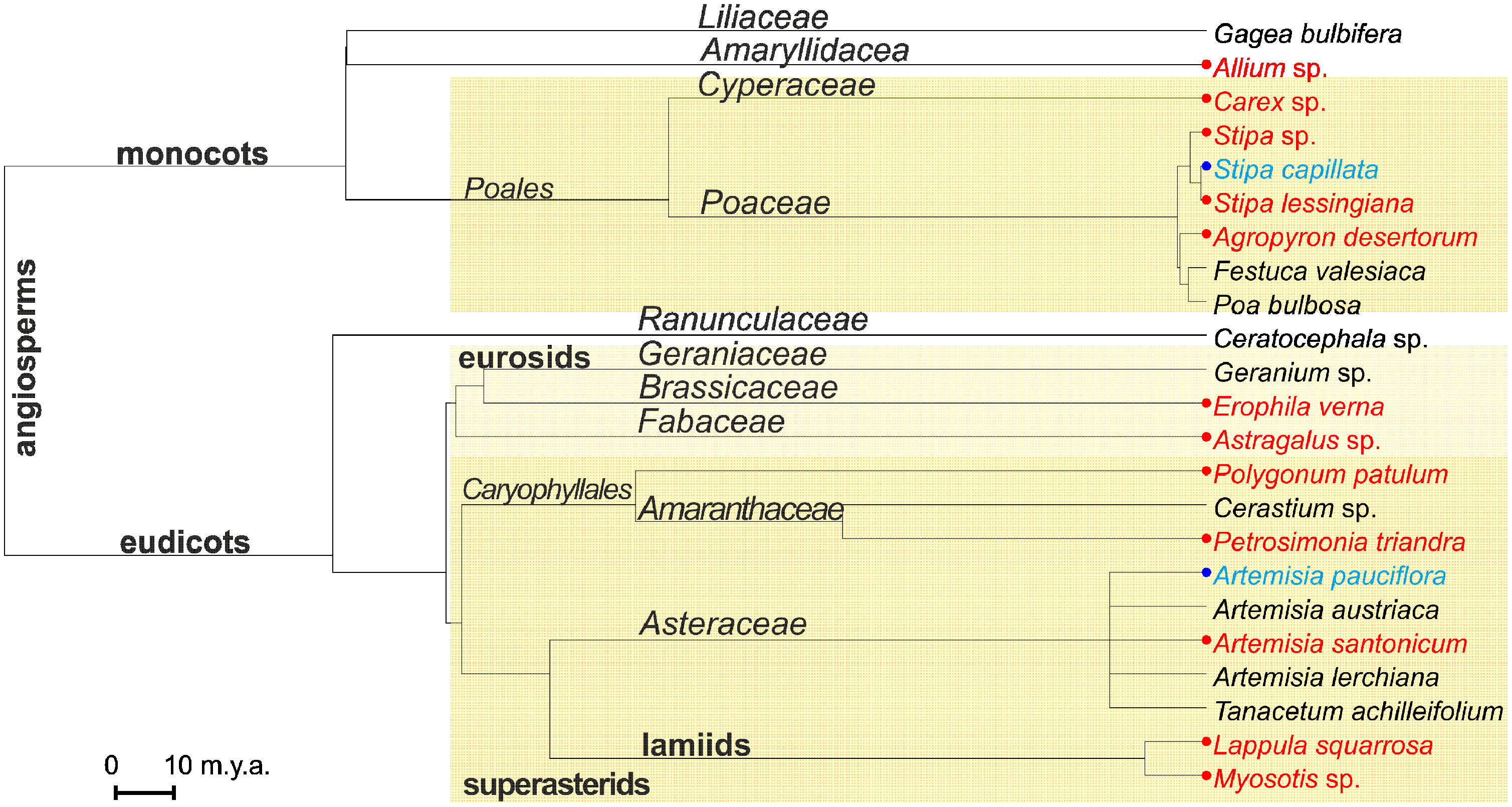

Supplement: Supplementary file 24 [file mmc24.jpg]

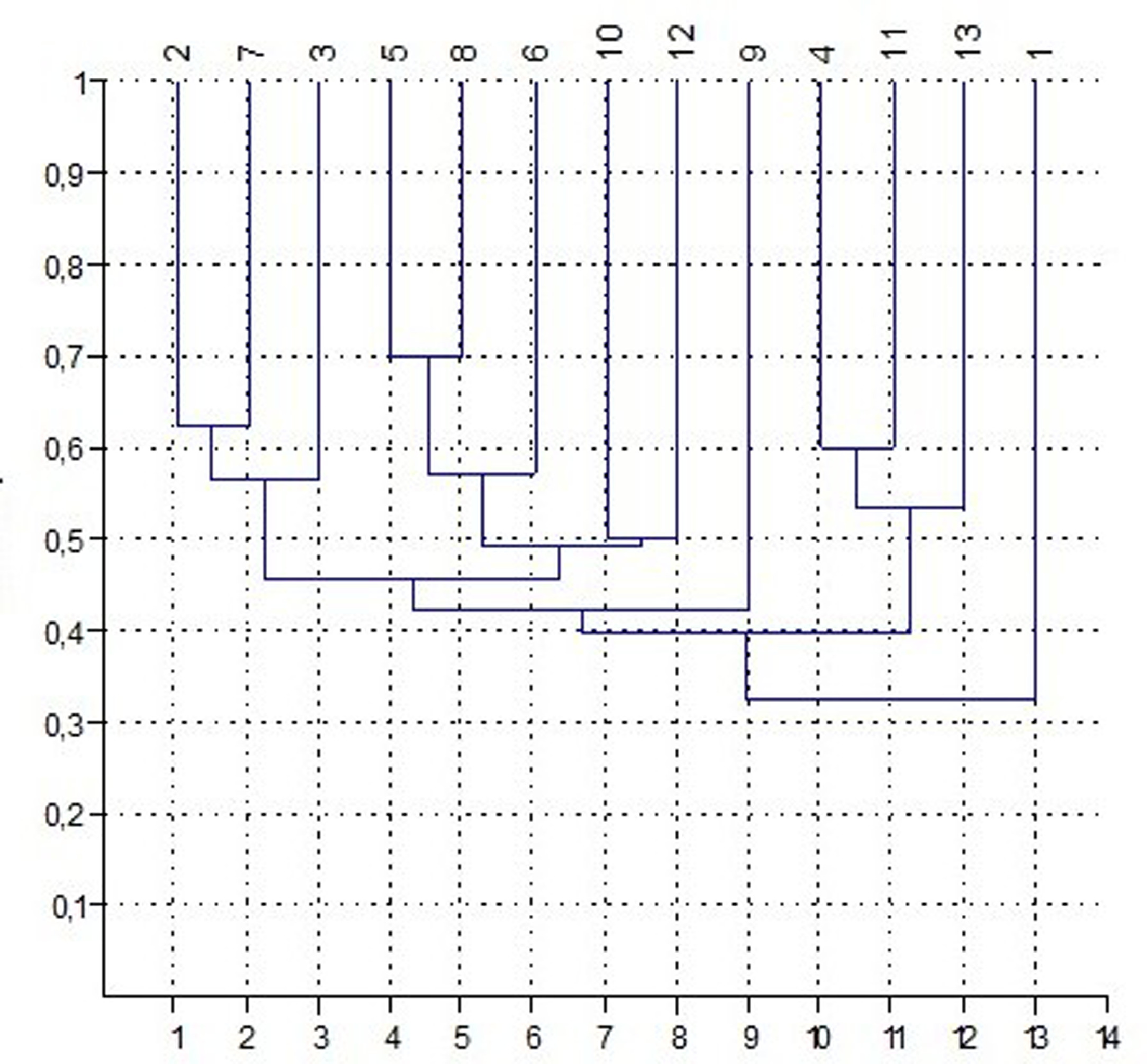

Supplement: Supplementary file 25 [file mmc25.jpg]

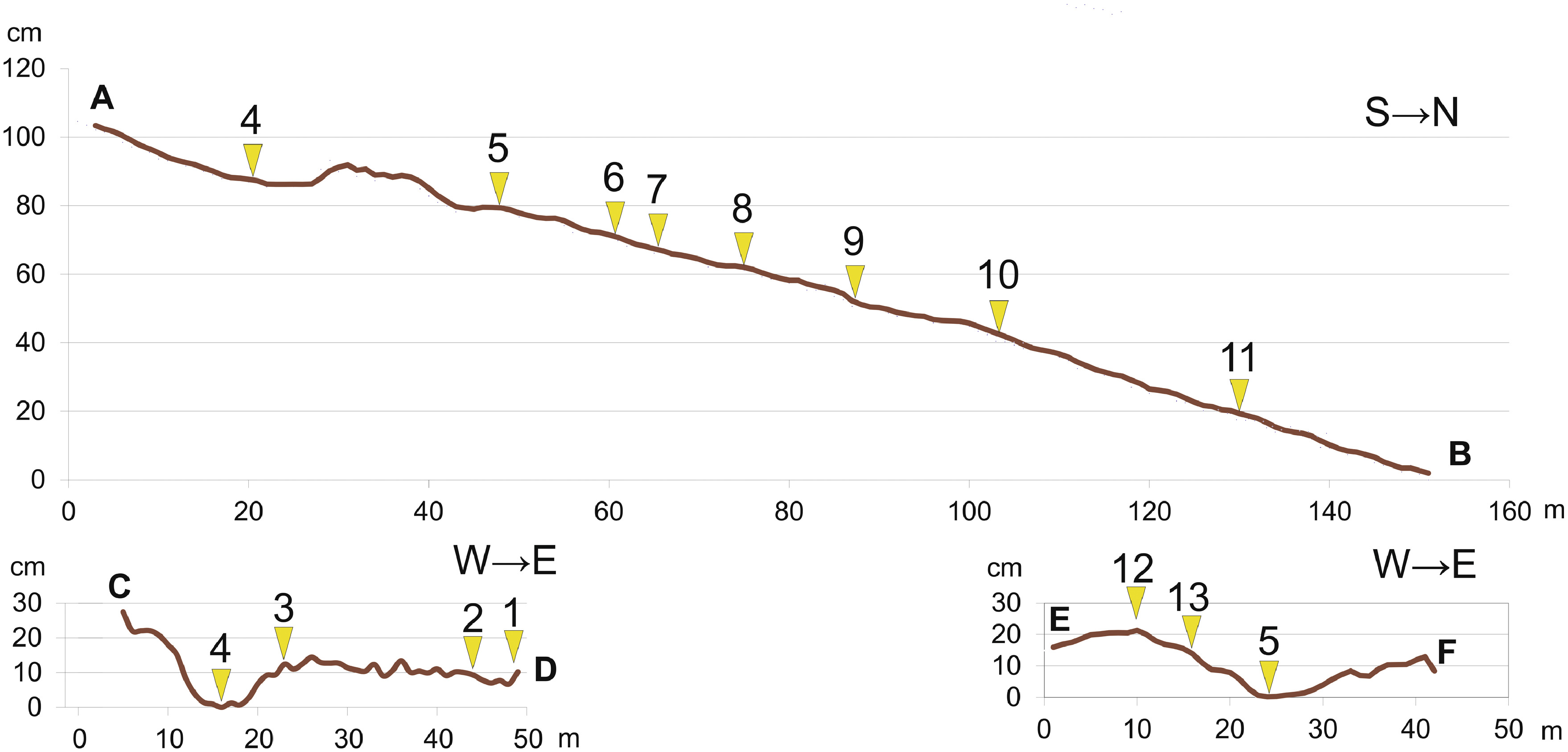

Supplement: Supplementary file 26 [file mmc26.jpg]
